# Supplementary material for: Transcriptome Analysis Reveals Vimentin-Induced Disruption of Cell–Cell Associations Augments Breast Cancer Cell Migration
Source: Cells. 2022 Dec 13;11(24):4035. doi: 10.3390/cells11244035 (PMC9776984; doi:10.3390/cells11244035)
Supplement: Supplementary file 1 [file cells-11-04035-s001.zip › DEGs and enrichment analysis.zip!/DEGs and enrichment analysis/WT_VIMvsVector KEGG/ALL/WT_VIMvsVector_ALL.pathway_map.zip!/WT_VIMvsVector_ALL.pathway_map/pathway/WT_VIMvsVector.html]

WT\_VIMvsVector.utf8.md


###WT\_VIMvsVector KEGG通路富集列表 \*\*\*

| KEGGID | Description | GeneRatio | BgRatio | pvalue | padj | geneID | geneName | keggID | Count | Up | Up\_Gene\_id | Down | Down\_Gene\_id |
| --- | --- | --- | --- | --- | --- | --- | --- | --- | --- | --- | --- | --- | --- |
| hsa03030 | DNA replication | 9\|253 | 41\|9228 | 0.0000012 | 0.0003210 | ENSG00000073111/ENSG00000100297/ENSG00000166508/ENSG00000168496/ENSG00000049541/ENSG00000062822/ENSG00000106628/ENSG00000014138/ENSG00000198056 | MCM2/MCM5/MCM7/FEN1/RFC2/POLD1/POLD2/POLA2/PRIM1 | hsa:4171/hsa:4174/hsa:4176/hsa:2237/hsa:5982/hsa:5424/hsa:5425/hsa:23649/hsa:5557 | 9 | 9 | ENSG00000073111/ENSG00000100297/ENSG00000166508/ENSG00000168496/ENSG00000049541/ENSG00000062822/ENSG00000106628/ENSG00000014138/ENSG00000198056 | 0 |  |
| hsa04110 | Cell cycle | 16\|253 | 163\|9228 | 0.0000096 | 0.0012482 | novel.507/ENSG00000093009/ENSG00000127564/ENSG00000166851/ENSG00000117399/ENSG00000073111/ENSG00000092969/ENSG00000085840/ENSG00000135476/ENSG00000100297/ENSG00000166508/ENSG00000101412/ENSG00000145386/ENSG00000164045/ENSG00000157456/ENSG00000116670 | -/CDC45/PKMYT1/PLK1/CDC20/MCM2/TGFB2/ORC1/ESPL1/MCM5/MCM7/E2F1/CCNA2/CDC25A/CCNB2/MAD2L2 | hsa:1870/hsa:8318/hsa:9088/hsa:5347/hsa:991/hsa:4171/hsa:7042/hsa:4998/hsa:9700/hsa:4174/hsa:4176/hsa:1869/hsa:890/hsa:993/hsa:9133/hsa:10459 | 16 | 15 | novel.507/ENSG00000093009/ENSG00000127564/ENSG00000166851/ENSG00000117399/ENSG00000073111/ENSG00000085840/ENSG00000135476/ENSG00000100297/ENSG00000166508/ENSG00000101412/ENSG00000145386/ENSG00000164045/ENSG00000157456/ENSG00000116670 | 1 | ENSG00000092969 |
| hsa05146 | Amoebiasis | 11\|253 | 94\|9228 | 0.0000489 | 0.0042374 | ENSG00000169429/ENSG00000058085/ENSG00000092969/ENSG00000188153/ENSG00000169031/ENSG00000164742/ENSG00000081041/ENSG00000135862/ENSG00000053747/ENSG00000137462/ENSG00000168542 | CXCL8/LAMC2/TGFB2/COL4A5/COL4A3/ADCY1/CXCL2/LAMC1/LAMA3/TLR2/COL3A1 | hsa:3576/hsa:3918/hsa:7042/hsa:1287/hsa:1285/hsa:107/hsa:2920/hsa:3915/hsa:3909/hsa:7097/hsa:1281 | 11 | 2 | ENSG00000164742/ENSG00000168542 | 9 | ENSG00000169429/ENSG00000058085/ENSG00000092969/ENSG00000188153/ENSG00000169031/ENSG00000081041/ENSG00000135862/ENSG00000053747/ENSG00000137462 |
| hsa05222 | Small cell lung cancer | 11\|253 | 121\|9228 | 0.0004716 | 0.0306523 | novel.507/ENSG00000005961/ENSG00000058085/ENSG00000188153/ENSG00000169031/ENSG00000101412/ENSG00000171791/ENSG00000173207/ENSG00000135862/ENSG00000053747/ENSG00000023445 | -/ITGA2B/LAMC2/COL4A5/COL4A3/E2F1/BCL2/CKS1B/LAMC1/LAMA3/BIRC3 | hsa:1870/hsa:3674/hsa:3918/hsa:1287/hsa:1285/hsa:1869/hsa:596/hsa:1163/hsa:3915/hsa:3909/hsa:330 | 11 | 5 | novel.507/ENSG00000005961/ENSG00000101412/ENSG00000171791/ENSG00000173207 | 6 | ENSG00000058085/ENSG00000188153/ENSG00000169031/ENSG00000135862/ENSG00000053747/ENSG00000023445 |
| hsa04080 | Neuroactive ligand-receptor interaction | 14\|253 | 219\|9228 | 0.0027901 | 0.1450866 | ENSG00000078401/ENSG00000164128/ENSG00000135346/ENSG00000136928/ENSG00000187730/ENSG00000109255/ENSG00000168539/ENSG00000069482/ENSG00000148926/ENSG00000168398/ENSG00000171631/ENSG00000065325/ENSG00000180739/ENSG00000164418 | EDN1/NPY1R/CGA/GABBR2/GABRD/NMU/CHRM1/GAL/ADM/BDKRB2/P2RY6/GLP2R/S1PR5/GRIK2 | hsa:1906/hsa:4886/hsa:1081/hsa:9568/hsa:2563/hsa:10874/hsa:1128/hsa:51083/hsa:133/hsa:624/hsa:5031/hsa:9340/hsa:53637/hsa:2898 | 14 | 7 | ENSG00000164128/ENSG00000187730/ENSG00000109255/ENSG00000168539/ENSG00000069482/ENSG00000171631/ENSG00000180739 | 7 | ENSG00000078401/ENSG00000135346/ENSG00000136928/ENSG00000148926/ENSG00000168398/ENSG00000065325/ENSG00000164418 |
| hsa05161 | Hepatitis B | 12\|253 | 179\|9228 | 0.0037516 | 0.1625701 | novel.507/ENSG00000157613/ENSG00000179388/ENSG00000169429/ENSG00000092969/ENSG00000138378/ENSG00000089685/ENSG00000101412/ENSG00000171791/ENSG00000145386/novel.600/ENSG00000137462 | -/CREB3L1/EGR3/CXCL8/TGFB2/STAT4/BIRC5/E2F1/BCL2/CCNA2/-/TLR2 | hsa:1870/hsa:90993/hsa:1960/hsa:3576/hsa:7042/hsa:6775/hsa:332/hsa:1869/hsa:596/hsa:890/hsa:1388/hsa:7097 | 12 | 7 | novel.507/ENSG00000179388/ENSG00000089685/ENSG00000101412/ENSG00000171791/ENSG00000145386/novel.600 | 5 | ENSG00000157613/ENSG00000169429/ENSG00000092969/ENSG00000138378/ENSG00000137462 |
| hsa04218 | Cellular senescence | 12\|253 | 204\|9228 | 0.0103704 | 0.3037142 | novel.507/ENSG00000169429/ENSG00000146674/ENSG00000111206/ENSG00000101057/ENSG00000162909/ENSG00000092969/ENSG00000101412/ENSG00000145386/ENSG00000164045/ENSG00000185650/ENSG00000157456 | -/CXCL8/IGFBP3/FOXM1/MYBL2/CAPN2/TGFB2/E2F1/CCNA2/CDC25A/ZFP36L1/CCNB2 | hsa:1870/hsa:3576/hsa:3486/hsa:2305/hsa:4605/hsa:824/hsa:7042/hsa:1869/hsa:890/hsa:993/hsa:677/hsa:9133 | 12 | 7 | novel.507/ENSG00000111206/ENSG00000101057/ENSG00000101412/ENSG00000145386/ENSG00000164045/ENSG00000157456 | 5 | ENSG00000169429/ENSG00000146674/ENSG00000162909/ENSG00000092969/ENSG00000185650 |
| hsa04926 | Relaxin signaling pathway | 9\|253 | 133\|9228 | 0.0108029 | 0.3037142 | ENSG00000078401/ENSG00000157613/ENSG00000127588/ENSG00000188153/ENSG00000169031/ENSG00000137745/ENSG00000164742/novel.600/ENSG00000168542 | EDN1/CREB3L1/GNG13/COL4A5/COL4A3/MMP13/ADCY1/-/COL3A1 | hsa:1906/hsa:90993/hsa:51764/hsa:1287/hsa:1285/hsa:4322/hsa:107/hsa:1388/hsa:1281 | 9 | 4 | ENSG00000127588/ENSG00000164742/novel.600/ENSG00000168542 | 5 | ENSG00000078401/ENSG00000157613/ENSG00000188153/ENSG00000169031/ENSG00000137745 |
| hsa04115 | p53 signaling pathway | 7\|253 | 89\|9228 | 0.0109470 | 0.3037142 | ENSG00000146674/ENSG00000177519/ENSG00000171791/ENSG00000075218/ENSG00000206075/ENSG00000184990/ENSG00000157456 | IGFBP3/RPRM/BCL2/GTSE1/SERPINB5/SIVA1/CCNB2 | hsa:3486/hsa:56475/hsa:596/hsa:51512/hsa:5268/hsa:10572/hsa:9133 | 7 | 5 | ENSG00000177519/ENSG00000171791/ENSG00000075218/ENSG00000184990/ENSG00000157456 | 2 | ENSG00000146674/ENSG00000206075 |
| hsa04512 | ECM-receptor interaction | 7\|253 | 91\|9228 | 0.0122837 | 0.3037142 | ENSG00000005961/ENSG00000058085/ENSG00000188153/ENSG00000169031/ENSG00000135862/ENSG00000053747/ENSG00000115221 | ITGA2B/LAMC2/COL4A5/COL4A3/LAMC1/LAMA3/ITGB6 | hsa:3674/hsa:3918/hsa:1287/hsa:1285/hsa:3915/hsa:3909/hsa:3694 | 7 | 1 | ENSG00000005961 | 6 | ENSG00000058085/ENSG00000188153/ENSG00000169031/ENSG00000135862/ENSG00000053747/ENSG00000115221 |
| hsa05206 | MicroRNAs in cancer | 11\|253 | 185\|9228 | 0.0128494 | 0.3037142 | ENSG00000026025/novel.507/ENSG00000092969/ENSG00000101412/ENSG00000171791/ENSG00000146670/ENSG00000164045/ENSG00000206075/ENSG00000277443/ENSG00000156103/ENSG00000088305 | VIM/-/TGFB2/E2F1/BCL2/CDCA5/CDC25A/SERPINB5/MARCKS/MMP16/DNMT3B | hsa:7431/hsa:1870/hsa:7042/hsa:1869/hsa:596/hsa:113130/hsa:993/hsa:5268/hsa:4082/hsa:4325/hsa:1789 | 11 | 8 | ENSG00000026025/novel.507/ENSG00000101412/ENSG00000171791/ENSG00000146670/ENSG00000164045/ENSG00000156103/ENSG00000088305 | 3 | ENSG00000092969/ENSG00000206075/ENSG00000277443 |
| hsa04151 | PI3K-Akt signaling pathway | 18\|253 | 376\|9228 | 0.0152243 | 0.3298591 | ENSG00000005961/ENSG00000157613/ENSG00000160867/ENSG00000058085/ENSG00000128052/ENSG00000127588/ENSG00000109321/ENSG00000188153/ENSG00000168539/ENSG00000169031/ENSG00000171791/novel.600/ENSG00000135862/ENSG00000053747/ENSG00000115221/ENSG00000037280/ENSG00000137462/ENSG00000118513 | ITGA2B/CREB3L1/FGFR4/LAMC2/KDR/GNG13/AREG/COL4A5/CHRM1/COL4A3/BCL2/-/LAMC1/LAMA3/ITGB6/FLT4/TLR2/MYB | hsa:3674/hsa:90993/hsa:2264/hsa:3918/hsa:3791/hsa:51764/hsa:374/hsa:1287/hsa:1128/hsa:1285/hsa:596/hsa:1388/hsa:3915/hsa:3909/hsa:3694/hsa:2324/hsa:7097/hsa:4602 | 18 | 8 | ENSG00000005961/ENSG00000160867/ENSG00000127588/ENSG00000168539/ENSG00000171791/novel.600/ENSG00000037280/ENSG00000118513 | 10 | ENSG00000157613/ENSG00000058085/ENSG00000128052/ENSG00000109321/ENSG00000188153/ENSG00000169031/ENSG00000135862/ENSG00000053747/ENSG00000115221/ENSG00000137462 |
| hsa04914 | Progesterone-mediated oocyte maturation | 8\|253 | 120\|9228 | 0.0172006 | 0.3440111 | ENSG00000082175/ENSG00000127564/ENSG00000166851/ENSG00000145386/ENSG00000164045/ENSG00000164742/ENSG00000157456/ENSG00000116670 | PGR/PKMYT1/PLK1/CCNA2/CDC25A/ADCY1/CCNB2/MAD2L2 | hsa:5241/hsa:9088/hsa:5347/hsa:890/hsa:993/hsa:107/hsa:9133/hsa:10459 | 8 | 8 | ENSG00000082175/ENSG00000127564/ENSG00000166851/ENSG00000145386/ENSG00000164045/ENSG00000164742/ENSG00000157456/ENSG00000116670 | 0 |  |
| hsa00830 | Retinol metabolism | 5\|253 | 57\|9228 | 0.0195689 | 0.3634219 | ENSG00000162496/ENSG00000128918/ENSG00000003137/ENSG00000256612/ENSG00000095596 | DHRS3/ALDH1A2/CYP26B1/CYP2B7P/CYP26A1 | hsa:9249/hsa:8854/hsa:56603/hsa:1555/hsa:1592 | 5 | 0 |  | 5 | ENSG00000162496/ENSG00000128918/ENSG00000003137/ENSG00000256612/ENSG00000095596 |
| hsa04933 | AGE-RAGE signaling pathway in diabetic complications | 7\|253 | 102\|9228 | 0.0218016 | 0.3778936 | ENSG00000078401/ENSG00000169429/ENSG00000092969/ENSG00000188153/ENSG00000169031/ENSG00000171791/ENSG00000168542 | EDN1/CXCL8/TGFB2/COL4A5/COL4A3/BCL2/COL3A1 | hsa:1906/hsa:3576/hsa:7042/hsa:1287/hsa:1285/hsa:596/hsa:1281 | 7 | 2 | ENSG00000171791/ENSG00000168542 | 5 | ENSG00000078401/ENSG00000169429/ENSG00000092969/ENSG00000188153/ENSG00000169031 |
| hsa04974 | Protein digestion and absorption | 6\|253 | 85\|9228 | 0.0289284 | 0.4417628 | ENSG00000092068/ENSG00000188153/ENSG00000164626/ENSG00000169031/ENSG00000104783/ENSG00000168542 | SLC7A8/COL4A5/KCNK5/COL4A3/KCNN4/COL3A1 | hsa:23428/hsa:1287/hsa:8645/hsa:1285/hsa:3783/hsa:1281 | 6 | 4 | ENSG00000092068/ENSG00000164626/ENSG00000104783/ENSG00000168542 | 2 | ENSG00000188153/ENSG00000169031 |
| hsa00340 | Histidine metabolism | 3\|253 | 25\|9228 | 0.0300150 | 0.4417628 | ENSG00000259583/ENSG00000184254/ENSG00000137124 | AC015712.2/ALDH1A3/ALDH1B1 | hsa:220/hsa:220/hsa:219 | 3 | 1 | ENSG00000137124 | 2 | ENSG00000259583/ENSG00000184254 |
| hsa00982 | Drug metabolism - cytochrome P450 | 5\|253 | 64\|9228 | 0.0305836 | 0.4417628 | ENSG00000213366/ENSG00000259583/ENSG00000184254/ENSG00000256612/ENSG00000241720 | GSTM2/AC015712.2/ALDH1A3/CYP2B7P/AL158847.1 | hsa:2946/hsa:220/hsa:220/hsa:1555/hsa:2947 | 5 | 0 |  | 5 | ENSG00000213366/ENSG00000259583/ENSG00000184254/ENSG00000256612/ENSG00000241720 |
| hsa04928 | Parathyroid hormone synthesis, secretion and action | 7\|253 | 113\|9228 | 0.0355174 | 0.4753502 | ENSG00000157613/ENSG00000137745/ENSG00000171791/ENSG00000164742/novel.600/ENSG00000109062/ENSG00000156103 | CREB3L1/MMP13/BCL2/ADCY1/-/SLC9A3R1/MMP16 | hsa:90993/hsa:4322/hsa:596/hsa:107/hsa:1388/hsa:9368/hsa:4325 | 7 | 5 | ENSG00000171791/ENSG00000164742/novel.600/ENSG00000109062/ENSG00000156103 | 2 | ENSG00000157613/ENSG00000137745 |
| hsa00240 | Pyrimidine metabolism | 5\|253 | 68\|9228 | 0.0383315 | 0.4753502 | ENSG00000168032/ENSG00000167900/ENSG00000128951/ENSG00000176890/ENSG00000168393 | ENTPD3/TK1/DUT/TYMS/DTYMK | hsa:956/hsa:7083/hsa:1854/hsa:7298/hsa:1841 | 5 | 4 | ENSG00000167900/ENSG00000128951/ENSG00000176890/ENSG00000168393 | 1 | ENSG00000168032 |
| hsa00980 | Metabolism of xenobiotics by cytochrome P450 | 5\|253 | 69\|9228 | 0.0404394 | 0.4753502 | ENSG00000213366/ENSG00000259583/ENSG00000184254/ENSG00000256612/ENSG00000241720 | GSTM2/AC015712.2/ALDH1A3/CYP2B7P/AL158847.1 | hsa:2946/hsa:220/hsa:220/hsa:1555/hsa:2947 | 5 | 0 |  | 5 | ENSG00000213366/ENSG00000259583/ENSG00000184254/ENSG00000256612/ENSG00000241720 |
| hsa04510 | Focal adhesion | 12\|253 | 249\|9228 | 0.0413073 | 0.4753502 | ENSG00000005961/ENSG00000058085/ENSG00000128052/ENSG00000162909/ENSG00000188153/ENSG00000169031/ENSG00000171791/ENSG00000135862/ENSG00000053747/ENSG00000023445/ENSG00000115221/ENSG00000037280 | ITGA2B/LAMC2/KDR/CAPN2/COL4A5/COL4A3/BCL2/LAMC1/LAMA3/BIRC3/ITGB6/FLT4 | hsa:3674/hsa:3918/hsa:3791/hsa:824/hsa:1287/hsa:1285/hsa:596/hsa:3915/hsa:3909/hsa:330/hsa:3694/hsa:2324 | 12 | 3 | ENSG00000005961/ENSG00000171791/ENSG00000037280 | 9 | ENSG00000058085/ENSG00000128052/ENSG00000162909/ENSG00000188153/ENSG00000169031/ENSG00000135862/ENSG00000053747/ENSG00000023445/ENSG00000115221 |
| hsa04621 | NOD-like receptor signaling pathway | 10\|253 | 195\|9228 | 0.0420502 | 0.4753502 | ENSG00000169429/ENSG00000117226/ENSG00000162645/ENSG00000171791/ENSG00000139112/ENSG00000111335/ENSG00000081041/ENSG00000023445/ENSG00000132357/ENSG00000187796 | CXCL8/GBP3/GBP2/BCL2/GABARAPL1/OAS2/CXCL2/BIRC3/CARD6/CARD9 | hsa:3576/hsa:2635/hsa:2634/hsa:596/hsa:23710/hsa:4939/hsa:2920/hsa:330/hsa:84674/hsa:64170 | 10 | 2 | ENSG00000171791/ENSG00000187796 | 8 | ENSG00000169429/ENSG00000117226/ENSG00000162645/ENSG00000139112/ENSG00000111335/ENSG00000081041/ENSG00000023445/ENSG00000132357 |
| hsa03410 | Base excision repair | 4\|253 | 51\|9228 | 0.0505206 | 0.4851263 | ENSG00000168496/ENSG00000065057/ENSG00000062822/ENSG00000106628 | FEN1/NTHL1/POLD1/POLD2 | hsa:2237/hsa:4913/hsa:5424/hsa:5425 | 4 | 4 | ENSG00000168496/ENSG00000065057/ENSG00000062822/ENSG00000106628 | 0 |  |
| hsa05323 | Rheumatoid arthritis | 5\|253 | 75\|9228 | 0.0545463 | 0.4851263 | ENSG00000107562/ENSG00000169429/ENSG00000092969/ENSG00000081041/ENSG00000137462 | CXCL12/CXCL8/TGFB2/CXCL2/TLR2 | hsa:6387/hsa:3576/hsa:7042/hsa:2920/hsa:7097 | 5 | 1 | ENSG00000107562 | 4 | ENSG00000169429/ENSG00000092969/ENSG00000081041/ENSG00000137462 |
| hsa04950 | Maturity onset diabetes of the young | 2\|253 | 14\|9228 | 0.0548530 | 0.4851263 | ENSG00000164749/ENSG00000116833 | HNF4G/NR5A2 | hsa:3174/hsa:2494 | 2 | 1 | ENSG00000116833 | 1 | ENSG00000164749 |
| hsa05166 | Human T-cell leukemia virus 1 infection | 12\|253 | 261\|9228 | 0.0554217 | 0.4851263 | novel.507/ENSG00000157613/ENSG00000117399/ENSG00000092969/ENSG00000135476/ENSG00000101412/ENSG00000145386/ENSG00000164742/ENSG00000117394/novel.600/ENSG00000164362/ENSG00000157456 | -/CREB3L1/CDC20/TGFB2/ESPL1/E2F1/CCNA2/ADCY1/SLC2A1/-/TERT/CCNB2 | hsa:1870/hsa:90993/hsa:991/hsa:7042/hsa:9700/hsa:1869/hsa:890/hsa:107/hsa:6513/hsa:1388/hsa:7015/hsa:9133 | 12 | 10 | novel.507/ENSG00000117399/ENSG00000135476/ENSG00000101412/ENSG00000145386/ENSG00000164742/ENSG00000117394/novel.600/ENSG00000164362/ENSG00000157456 | 2 | ENSG00000157613/ENSG00000092969 |
| hsa05145 | Toxoplasmosis | 7\|253 | 125\|9228 | 0.0559391 | 0.4851263 | ENSG00000058085/ENSG00000092969/ENSG00000171791/ENSG00000135862/ENSG00000053747/ENSG00000023445/ENSG00000137462 | LAMC2/TGFB2/BCL2/LAMC1/LAMA3/BIRC3/TLR2 | hsa:3918/hsa:7042/hsa:596/hsa:3915/hsa:3909/hsa:330/hsa:7097 | 7 | 1 | ENSG00000171791 | 6 | ENSG00000058085/ENSG00000092969/ENSG00000135862/ENSG00000053747/ENSG00000023445/ENSG00000137462 |
| hsa04915 | Estrogen signaling pathway | 9\|253 | 180\|9228 | 0.0594010 | 0.4851263 | ENSG00000082175/ENSG00000164850/ENSG00000157613/ENSG00000136928/ENSG00000160182/ENSG00000131759/ENSG00000171791/ENSG00000164742/novel.600 | PGR/GPER1/CREB3L1/GABBR2/TFF1/RARA/BCL2/ADCY1/- | hsa:5241/hsa:2852/hsa:90993/hsa:9568/hsa:7031/hsa:5914/hsa:596/hsa:107/hsa:1388 | 9 | 7 | ENSG00000082175/ENSG00000164850/ENSG00000160182/ENSG00000131759/ENSG00000171791/ENSG00000164742/novel.600 | 2 | ENSG00000157613/ENSG00000136928 |
| hsa01523 | Antifolate resistance | 3\|253 | 33\|9228 | 0.0607688 | 0.4851263 | ENSG00000125257/ENSG00000176890/ENSG00000173638 | ABCC4/TYMS/SLC19A1 | hsa:10257/hsa:7298/hsa:6573 | 3 | 2 | ENSG00000176890/ENSG00000173638 | 1 | ENSG00000125257 |
| hsa05144 | Malaria | 3\|253 | 33\|9228 | 0.0607688 | 0.4851263 | ENSG00000169429/ENSG00000092969/ENSG00000137462 | CXCL8/TGFB2/TLR2 | hsa:3576/hsa:7042/hsa:7097 | 3 | 0 |  | 3 | ENSG00000169429/ENSG00000092969/ENSG00000137462 |
| hsa04727 | GABAergic synapse | 5\|253 | 78\|9228 | 0.0625436 | 0.4851263 | ENSG00000136928/ENSG00000127588/ENSG00000187730/ENSG00000139112/ENSG00000164742 | GABBR2/GNG13/GABRD/GABARAPL1/ADCY1 | hsa:9568/hsa:51764/hsa:2563/hsa:23710/hsa:107 | 5 | 3 | ENSG00000127588/ENSG00000187730/ENSG00000164742 | 2 | ENSG00000136928/ENSG00000139112 |
| hsa04911 | Insulin secretion | 5\|253 | 78\|9228 | 0.0625436 | 0.4851263 | ENSG00000157613/ENSG00000104783/ENSG00000164742/ENSG00000117394/novel.600 | CREB3L1/KCNN4/ADCY1/SLC2A1/- | hsa:90993/hsa:3783/hsa:107/hsa:6513/hsa:1388 | 5 | 4 | ENSG00000104783/ENSG00000164742/ENSG00000117394/novel.600 | 1 | ENSG00000157613 |
| hsa04923 | Regulation of lipolysis in adipocytes | 4\|253 | 55\|9228 | 0.0634396 | 0.4851263 | ENSG00000185532/ENSG00000164128/ENSG00000135346/ENSG00000164742 | PRKG1/NPY1R/CGA/ADCY1 | hsa:5592/hsa:4886/hsa:1081/hsa:107 | 4 | 2 | ENSG00000164128/ENSG00000164742 | 2 | ENSG00000185532/ENSG00000135346 |
| hsa00410 | beta-Alanine metabolism | 3\|253 | 34\|9228 | 0.0653517 | 0.4854701 | ENSG00000259583/ENSG00000184254/ENSG00000137124 | AC015712.2/ALDH1A3/ALDH1B1 | hsa:220/hsa:220/hsa:219 | 3 | 1 | ENSG00000137124 | 2 | ENSG00000259583/ENSG00000184254 |
| hsa00330 | Arginine and proline metabolism | 4\|253 | 58\|9228 | 0.0741587 | 0.5345457 | ENSG00000104524/ENSG00000130066/ENSG00000166165/ENSG00000137124 | PYCR3/SAT1/CKB/ALDH1B1 | hsa:65263/hsa:6303/hsa:1152/hsa:219 | 4 | 3 | ENSG00000104524/ENSG00000166165/ENSG00000137124 | 1 | ENSG00000130066 |
| hsa04725 | Cholinergic synapse | 6\|253 | 108\|9228 | 0.0760700 | 0.5345457 | ENSG00000157613/ENSG00000127588/ENSG00000168539/ENSG00000171791/ENSG00000164742/ENSG00000087085 | CREB3L1/GNG13/CHRM1/BCL2/ADCY1/ACHE | hsa:90993/hsa:51764/hsa:1128/hsa:596/hsa:107/hsa:43 | 6 | 4 | ENSG00000127588/ENSG00000168539/ENSG00000171791/ENSG00000164742 | 2 | ENSG00000157613/ENSG00000087085 |
| hsa04061 | Viral protein interaction with cytokine and cytokine receptor | 4\|253 | 61\|9228 | 0.0857331 | 0.5704242 | ENSG00000107562/ENSG00000169429/ENSG00000006606/ENSG00000081041 | CXCL12/CXCL8/CCL26/CXCL2 | hsa:6387/hsa:3576/hsa:10344/hsa:2920 | 4 | 1 | ENSG00000107562 | 3 | ENSG00000169429/ENSG00000006606/ENSG00000081041 |
| hsa00480 | Glutathione metabolism | 4\|253 | 62\|9228 | 0.0897758 | 0.5704242 | ENSG00000213366/ENSG00000167741/ENSG00000166825/ENSG00000241720 | GSTM2/GGT6/ANPEP/AL158847.1 | hsa:2946/hsa:124975/hsa:290/hsa:2947 | 4 | 2 | ENSG00000167741/ENSG00000166825 | 2 | ENSG00000213366/ENSG00000241720 |
| hsa00350 | Tyrosine metabolism | 3\|253 | 39\|9228 | 0.0905036 | 0.5704242 | ENSG00000259583/ENSG00000184254/ENSG00000180176 | AC015712.2/ALDH1A3/TH | hsa:220/hsa:220/hsa:7054 | 3 | 1 | ENSG00000180176 | 2 | ENSG00000259583/ENSG00000184254 |
| hsa03430 | Mismatch repair | 3\|253 | 39\|9228 | 0.0905036 | 0.5704242 | ENSG00000049541/ENSG00000062822/ENSG00000106628 | RFC2/POLD1/POLD2 | hsa:5982/hsa:5424/hsa:5425 | 3 | 3 | ENSG00000049541/ENSG00000062822/ENSG00000106628 | 0 |  |
| hsa01524 | Platinum drug resistance | 5\|253 | 88\|9228 | 0.0936857 | 0.5704242 | ENSG00000213366/ENSG00000089685/ENSG00000171791/ENSG00000023445/ENSG00000241720 | GSTM2/BIRC5/BCL2/BIRC3/AL158847.1 | hsa:2946/hsa:332/hsa:596/hsa:330/hsa:2947 | 5 | 2 | ENSG00000089685/ENSG00000171791 | 3 | ENSG00000213366/ENSG00000023445/ENSG00000241720 |
| hsa00360 | Phenylalanine metabolism | 2\|253 | 19\|9228 | 0.0943394 | 0.5704242 | ENSG00000259583/ENSG00000184254 | AC015712.2/ALDH1A3 | hsa:220/hsa:220 | 2 | 0 |  | 2 | ENSG00000259583/ENSG00000184254 |
| hsa04114 | Oocyte meiosis | 8\|253 | 171\|9228 | 0.0979927 | 0.5790479 | ENSG00000082175/ENSG00000127564/ENSG00000166851/ENSG00000117399/ENSG00000135476/ENSG00000164742/ENSG00000157456/ENSG00000116670 | PGR/PKMYT1/PLK1/CDC20/ESPL1/ADCY1/CCNB2/MAD2L2 | hsa:5241/hsa:9088/hsa:5347/hsa:991/hsa:9700/hsa:107/hsa:9133/hsa:10459 | 8 | 8 | ENSG00000082175/ENSG00000127564/ENSG00000166851/ENSG00000117399/ENSG00000135476/ENSG00000164742/ENSG00000157456/ENSG00000116670 | 0 |  |
| hsa04270 | Vascular smooth muscle contraction | 7\|253 | 146\|9228 | 0.1063820 | 0.5799036 | ENSG00000078401/ENSG00000185532/ENSG00000122679/ENSG00000122786/ENSG00000148926/ENSG00000164742/ENSG00000188257 | EDN1/PRKG1/RAMP3/CALD1/ADM/ADCY1/PLA2G2A | hsa:1906/hsa:5592/hsa:10268/hsa:800/hsa:133/hsa:107/hsa:5320 | 7 | 2 | ENSG00000122679/ENSG00000164742 | 5 | ENSG00000078401/ENSG00000185532/ENSG00000122786/ENSG00000148926/ENSG00000188257 |
| hsa04360 | Axon guidance | 9\|253 | 204\|9228 | 0.1083364 | 0.5799036 | ENSG00000107562/ENSG00000074527/ENSG00000157851/ENSG00000112902/ENSG00000167680/ENSG00000065320/ENSG00000196220/ENSG00000172602/ENSG00000044524 | CXCL12/NTN4/DPYSL5/SEMA5A/SEMA6B/NTN1/SRGAP3/RND1/EPHA3 | hsa:6387/hsa:59277/hsa:56896/hsa:9037/hsa:10501/hsa:9423/hsa:9901/hsa:27289/hsa:2042 | 9 | 5 | ENSG00000107562/ENSG00000157851/ENSG00000167680/ENSG00000065320/ENSG00000044524 | 4 | ENSG00000074527/ENSG00000112902/ENSG00000196220/ENSG00000172602 |
| hsa05142 | Chagas disease (American trypanosomiasis) | 5\|253 | 93\|9228 | 0.1117466 | 0.5799036 | ENSG00000169429/ENSG00000092969/ENSG00000164742/ENSG00000168398/ENSG00000137462 | CXCL8/TGFB2/ADCY1/BDKRB2/TLR2 | hsa:3576/hsa:7042/hsa:107/hsa:624/hsa:7097 | 5 | 1 | ENSG00000164742 | 4 | ENSG00000169429/ENSG00000092969/ENSG00000168398/ENSG00000137462 |
| hsa04977 | Vitamin digestion and absorption | 2\|253 | 21\|9228 | 0.1118572 | 0.5799036 | ENSG00000086205/ENSG00000173638 | FOLH1/SLC19A1 | hsa:2346/hsa:6573 | 2 | 1 | ENSG00000173638 | 1 | ENSG00000086205 |
| hsa04929 | GnRH secretion | 4\|253 | 68\|9228 | 0.1158743 | 0.5799036 | ENSG00000164850/ENSG00000135346/ENSG00000136928/ENSG00000104783 | GPER1/CGA/GABBR2/KCNN4 | hsa:2852/hsa:1081/hsa:9568/hsa:3783 | 4 | 2 | ENSG00000164850/ENSG00000104783 | 2 | ENSG00000135346/ENSG00000136928 |
| hsa05321 | Inflammatory bowel disease (IBD) | 3\|253 | 44\|9228 | 0.1190238 | 0.5799036 | ENSG00000092969/ENSG00000138378/ENSG00000137462 | TGFB2/STAT4/TLR2 | hsa:7042/hsa:6775/hsa:7097 | 3 | 0 |  | 3 | ENSG00000092969/ENSG00000138378/ENSG00000137462 |
| hsa04742 | Taste transduction | 3\|253 | 45\|9228 | 0.1250861 | 0.5799036 | ENSG00000136928/ENSG00000127588/ENSG00000164626 | GABBR2/GNG13/KCNK5 | hsa:9568/hsa:51764/hsa:8645 | 3 | 2 | ENSG00000127588/ENSG00000164626 | 1 | ENSG00000136928 |
| hsa05032 | Morphine addiction | 4\|253 | 70\|9228 | 0.1252376 | 0.5799036 | ENSG00000136928/ENSG00000127588/ENSG00000187730/ENSG00000164742 | GABBR2/GNG13/GABRD/ADCY1 | hsa:9568/hsa:51764/hsa:2563/hsa:107 | 4 | 3 | ENSG00000127588/ENSG00000187730/ENSG00000164742 | 1 | ENSG00000136928 |
| hsa04610 | Complement and coagulation cascades | 4\|253 | 71\|9228 | 0.1300361 | 0.5799036 | ENSG00000237248/ENSG00000175899/ENSG00000168398/ENSG00000131187 | LINC00987/A2M/BDKRB2/F12 | hsa:2/hsa:2/hsa:624/hsa:2161 | 4 | 1 | ENSG00000131187 | 3 | ENSG00000237248/ENSG00000175899/ENSG00000168398 |
| hsa04215 | Apoptosis - multiple species | 3\|253 | 46\|9228 | 0.1312575 | 0.5799036 | ENSG00000089685/ENSG00000171791/ENSG00000023445 | BIRC5/BCL2/BIRC3 | hsa:332/hsa:596/hsa:330 | 3 | 2 | ENSG00000089685/ENSG00000171791 | 1 | ENSG00000023445 |
| hsa04064 | NF-kappa B signaling pathway | 5\|253 | 98\|9228 | 0.1313553 | 0.5799036 | ENSG00000107562/ENSG00000169429/ENSG00000171791/ENSG00000081041/ENSG00000023445 | CXCL12/CXCL8/BCL2/CXCL2/BIRC3 | hsa:6387/hsa:3576/hsa:596/hsa:2920/hsa:330 | 5 | 2 | ENSG00000107562/ENSG00000171791 | 3 | ENSG00000169429/ENSG00000081041/ENSG00000023445 |
| hsa04024 | cAMP signaling pathway | 9\|253 | 214\|9228 | 0.1339108 | 0.5799036 | ENSG00000125257/ENSG00000078401/ENSG00000157613/ENSG00000164128/ENSG00000135346/ENSG00000136928/ENSG00000168539/ENSG00000125398/ENSG00000164742 | ABCC4/EDN1/CREB3L1/NPY1R/CGA/GABBR2/CHRM1/SOX9/ADCY1 | hsa:10257/hsa:1906/hsa:90993/hsa:4886/hsa:1081/hsa:9568/hsa:1128/hsa:6662/hsa:107 | 9 | 3 | ENSG00000164128/ENSG00000168539/ENSG00000164742 | 6 | ENSG00000125257/ENSG00000078401/ENSG00000157613/ENSG00000135346/ENSG00000136928/ENSG00000125398 |
| hsa05204 | Chemical carcinogenesis | 4\|253 | 72\|9228 | 0.1349101 | 0.5799036 | ENSG00000213366/ENSG00000259583/ENSG00000184254/ENSG00000241720 | GSTM2/AC015712.2/ALDH1A3/AL158847.1 | hsa:2946/hsa:220/hsa:220/hsa:2947 | 4 | 0 |  | 4 | ENSG00000213366/ENSG00000259583/ENSG00000184254/ENSG00000241720 |
| hsa01522 | Endocrine resistance | 5\|253 | 99\|9228 | 0.1354534 | 0.5799036 | ENSG00000164850/novel.507/ENSG00000101412/ENSG00000171791/ENSG00000164742 | GPER1/-/E2F1/BCL2/ADCY1 | hsa:2852/hsa:1870/hsa:1869/hsa:596/hsa:107 | 5 | 5 | ENSG00000164850/novel.507/ENSG00000101412/ENSG00000171791/ENSG00000164742 | 0 |  |
| hsa05030 | Cocaine addiction | 3\|253 | 47\|9228 | 0.1375338 | 0.5799036 | ENSG00000157613/ENSG00000180176/novel.600 | CREB3L1/TH/- | hsa:90993/hsa:7054/hsa:1388 | 3 | 2 | ENSG00000180176/novel.600 | 1 | ENSG00000157613 |
| hsa04514 | Cell adhesion molecules (CAMs) | 6\|253 | 128\|9228 | 0.1394390 | 0.5799036 | ENSG00000179776/ENSG00000173482/ENSG00000134258/ENSG00000107738/ENSG00000188404/ENSG00000105695 | CDH5/PTPRM/VTCN1/VSIR/SELL/MAG | hsa:1003/hsa:5797/hsa:79679/hsa:64115/hsa:6402/hsa:4099 | 6 | 1 | ENSG00000105695 | 5 | ENSG00000179776/ENSG00000173482/ENSG00000134258/ENSG00000107738/ENSG00000188404 |
| hsa04918 | Thyroid hormone synthesis | 4\|253 | 73\|9228 | 0.1398576 | 0.5799036 | ENSG00000157613/ENSG00000135346/ENSG00000164742/novel.600 | CREB3L1/CGA/ADCY1/- | hsa:90993/hsa:1081/hsa:107/hsa:1388 | 4 | 2 | ENSG00000164742/novel.600 | 2 | ENSG00000157613/ENSG00000135346 |
| hsa04976 | Bile secretion | 4\|253 | 73\|9228 | 0.1398576 | 0.5799036 | ENSG00000125257/ENSG00000080493/ENSG00000164742/ENSG00000117394 | ABCC4/SLC4A4/ADCY1/SLC2A1 | hsa:10257/hsa:8671/hsa:107/hsa:6513 | 4 | 2 | ENSG00000164742/ENSG00000117394 | 2 | ENSG00000125257/ENSG00000080493 |
| hsa03440 | Homologous recombination | 3\|253 | 48\|9228 | 0.1439106 | 0.5799036 | ENSG00000154920/ENSG00000062822/ENSG00000106628 | EME1/POLD1/POLD2 | hsa:146956/hsa:5424/hsa:5425 | 3 | 3 | ENSG00000154920/ENSG00000062822/ENSG00000106628 | 0 |  |
| hsa05219 | Bladder cancer | 3\|253 | 48\|9228 | 0.1439106 | 0.5799036 | novel.507/ENSG00000169429/ENSG00000101412 | -/CXCL8/E2F1 | hsa:1870/hsa:3576/hsa:1869 | 3 | 2 | novel.507/ENSG00000101412 | 1 | ENSG00000169429 |
| hsa05418 | Fluid shear stress and atherosclerosis | 8\|253 | 188\|9228 | 0.1449759 | 0.5799036 | ENSG00000179776/ENSG00000078401/ENSG00000005961/ENSG00000213366/ENSG00000128052/ENSG00000116701/ENSG00000171791/ENSG00000241720 | CDH5/EDN1/ITGA2B/GSTM2/KDR/NCF2/BCL2/AL158847.1 | hsa:1003/hsa:1906/hsa:3674/hsa:2946/hsa:3791/hsa:4688/hsa:596/hsa:2947 | 8 | 2 | ENSG00000005961/ENSG00000171791 | 6 | ENSG00000179776/ENSG00000078401/ENSG00000213366/ENSG00000128052/ENSG00000116701/ENSG00000241720 |
| hsa04211 | Longevity regulating pathway | 5\|253 | 103\|9228 | 0.1524005 | 0.5966182 | ENSG00000157613/ENSG00000109819/ENSG00000164742/novel.600/ENSG00000132170 | CREB3L1/PPARGC1A/ADCY1/-/PPARG | hsa:90993/hsa:10891/hsa:107/hsa:1388/hsa:5468 | 5 | 2 | ENSG00000164742/novel.600 | 3 | ENSG00000157613/ENSG00000109819/ENSG00000132170 |
| hsa05165 | Human papillomavirus infection | 14\|253 | 377\|9228 | 0.1537439 | 0.5966182 | ENSG00000135547/ENSG00000005961/ENSG00000157613/ENSG00000058085/ENSG00000188153/ENSG00000169031/ENSG00000101412/ENSG00000145386/ENSG00000131462/ENSG00000135862/ENSG00000109062/ENSG00000053747/ENSG00000115221/ENSG00000164362 | HEY2/ITGA2B/CREB3L1/LAMC2/COL4A5/COL4A3/E2F1/CCNA2/TUBG1/LAMC1/SLC9A3R1/LAMA3/ITGB6/TERT | hsa:23493/hsa:3674/hsa:90993/hsa:3918/hsa:1287/hsa:1285/hsa:1869/hsa:890/hsa:7283/hsa:3915/hsa:9368/hsa:3909/hsa:3694/hsa:7015 | 14 | 7 | ENSG00000135547/ENSG00000005961/ENSG00000101412/ENSG00000145386/ENSG00000131462/ENSG00000109062/ENSG00000164362 | 7 | ENSG00000157613/ENSG00000058085/ENSG00000188153/ENSG00000169031/ENSG00000135862/ENSG00000053747/ENSG00000115221 |
| hsa04540 | Gap junction | 5\|253 | 104\|9228 | 0.1567709 | 0.5994182 | ENSG00000185532/ENSG00000214222/ENSG00000123416/ENSG00000164742/ENSG00000104833 | PRKG1/TUBBP2/TUBA1B/ADCY1/TUBB4A | hsa:5592/hsa:203068/hsa:10376/hsa:107/hsa:10382 | 5 | 4 | ENSG00000214222/ENSG00000123416/ENSG00000164742/ENSG00000104833 | 1 | ENSG00000185532 |
| hsa02010 | ABC transporters | 3\|253 | 51\|9228 | 0.1635995 | 0.6164620 | ENSG00000125257/ENSG00000198691/ENSG00000144452 | ABCC4/ABCA4/ABCA12 | hsa:10257/hsa:24/hsa:26154 | 3 | 1 | ENSG00000144452 | 2 | ENSG00000125257/ENSG00000198691 |
| hsa00600 | Sphingolipid metabolism | 3\|253 | 52\|9228 | 0.1703341 | 0.6326697 | ENSG00000177076/ENSG00000168350/ENSG00000102393 | ACER2/DEGS2/GLA | hsa:340485/hsa:123099/hsa:2717 | 3 | 2 | ENSG00000168350/ENSG00000102393 | 1 | ENSG00000177076 |
| hsa00100 | Steroid biosynthesis | 2\|253 | 29\|9228 | 0.1882484 | 0.6893602 | ENSG00000167780/ENSG00000147155 | SOAT2/EBP | hsa:8435/hsa:10682 | 2 | 2 | ENSG00000167780/ENSG00000147155 | 0 |  |
| hsa03420 | Nucleotide excision repair | 3\|253 | 56\|9228 | 0.1980153 | 0.7150553 | ENSG00000049541/ENSG00000062822/ENSG00000106628 | RFC2/POLD1/POLD2 | hsa:5982/hsa:5424/hsa:5425 | 3 | 3 | ENSG00000049541/ENSG00000062822/ENSG00000106628 | 0 |  |
| hsa05202 | Transcriptional misregulation in cancer | 8\|253 | 205\|9228 | 0.2011119 | 0.7162888 | ENSG00000164120/ENSG00000169429/ENSG00000146674/ENSG00000131759/ENSG00000145386/ENSG00000023445/ENSG00000058091/ENSG00000132170 | HPGD/CXCL8/IGFBP3/RARA/CCNA2/BIRC3/CDK14/PPARG | hsa:3248/hsa:3576/hsa:3486/hsa:5914/hsa:890/hsa:330/hsa:5218/hsa:5468 | 8 | 2 | ENSG00000131759/ENSG00000145386 | 6 | ENSG00000164120/ENSG00000169429/ENSG00000146674/ENSG00000023445/ENSG00000058091/ENSG00000132170 |
| hsa00983 | Drug metabolism - other enzymes | 4\|253 | 91\|9228 | 0.2392376 | 0.8306413 | ENSG00000213366/ENSG00000167900/ENSG00000128951/ENSG00000241720 | GSTM2/TK1/DUT/AL158847.1 | hsa:2946/hsa:7083/hsa:1854/hsa:2947 | 4 | 2 | ENSG00000167900/ENSG00000128951 | 2 | ENSG00000213366/ENSG00000241720 |
| hsa00430 | Taurine and hypotaurine metabolism | 1\|253 | 10\|9228 | 0.2428028 | 0.8306413 | ENSG00000167741 | GGT6 | hsa:124975 | 1 | 1 | ENSG00000167741 | 0 |  |
| hsa00920 | Sulfur metabolism | 1\|253 | 10\|9228 | 0.2428028 | 0.8306413 | ENSG00000143416 | SELENBP1 | hsa:8991 | 1 | 1 | ENSG00000143416 | 0 |  |
| hsa00270 | Cysteine and methionine metabolism | 3\|253 | 63\|9228 | 0.2486755 | 0.8396836 | ENSG00000151224/ENSG00000088305/ENSG00000092621 | MAT1A/DNMT3B/PHGDH | hsa:4143/hsa:1789/hsa:26227 | 3 | 3 | ENSG00000151224/ENSG00000088305/ENSG00000092621 | 0 |  |
| hsa04713 | Circadian entrainment | 4\|253 | 94\|9228 | 0.2572296 | 0.8467426 | ENSG00000185532/ENSG00000108551/ENSG00000127588/ENSG00000164742 | PRKG1/RASD1/GNG13/ADCY1 | hsa:5592/hsa:51655/hsa:51764/hsa:107 | 4 | 2 | ENSG00000127588/ENSG00000164742 | 2 | ENSG00000185532/ENSG00000108551 |
| hsa04927 | Cortisol synthesis and secretion | 3\|253 | 65\|9228 | 0.2635136 | 0.8467426 | ENSG00000157613/ENSG00000164742/novel.600 | CREB3L1/ADCY1/- | hsa:90993/hsa:107/hsa:1388 | 3 | 2 | ENSG00000164742/novel.600 | 1 | ENSG00000157613 |
| hsa05031 | Amphetamine addiction | 3\|253 | 65\|9228 | 0.2635136 | 0.8467426 | ENSG00000157613/ENSG00000180176/novel.600 | CREB3L1/TH/- | hsa:90993/hsa:7054/hsa:1388 | 3 | 2 | ENSG00000180176/novel.600 | 1 | ENSG00000157613 |
| hsa04668 | TNF signaling pathway | 5\|253 | 126\|9228 | 0.2637929 | 0.8467426 | ENSG00000078401/ENSG00000157613/ENSG00000081041/novel.600/ENSG00000023445 | EDN1/CREB3L1/CXCL2/-/BIRC3 | hsa:1906/hsa:90993/hsa:2920/hsa:1388/hsa:330 | 5 | 1 | novel.600 | 4 | ENSG00000078401/ENSG00000157613/ENSG00000081041/ENSG00000023445 |
| hsa00561 | Glycerolipid metabolism | 3\|253 | 67\|9228 | 0.2784587 | 0.8829177 | ENSG00000101670/ENSG00000137124/ENSG00000102393 | LIPG/ALDH1B1/GLA | hsa:9388/hsa:219/hsa:2717 | 3 | 2 | ENSG00000137124/ENSG00000102393 | 1 | ENSG00000101670 |
| hsa00072 | Synthesis and degradation of ketone bodies | 1\|253 | 12\|9228 | 0.2837992 | 0.8890094 | ENSG00000134240 | HMGCS2 | hsa:3158 | 1 | 0 |  | 1 | ENSG00000134240 |
| hsa04216 | Ferroptosis | 3\|253 | 69\|9228 | 0.2934828 | 0.8959166 | ENSG00000047457/ENSG00000130066/ENSG00000240216 | CP/SAT1/CPHL1P | hsa:1356/hsa:6303/hsa:1356 | 3 | 0 |  | 3 | ENSG00000047457/ENSG00000130066/ENSG00000240216 |
| hsa04071 | Sphingolipid signaling pathway | 5\|253 | 132\|9228 | 0.2955843 | 0.8959166 | ENSG00000177076/ENSG00000168350/ENSG00000171791/ENSG00000168398/ENSG00000180739 | ACER2/DEGS2/BCL2/BDKRB2/S1PR5 | hsa:340485/hsa:123099/hsa:596/hsa:624/hsa:53637 | 5 | 3 | ENSG00000168350/ENSG00000171791/ENSG00000180739 | 2 | ENSG00000177076/ENSG00000168398 |
| hsa05169 | Epstein-Barr virus infection | 8\|253 | 230\|9228 | 0.2963416 | 0.8959166 | ENSG00000026025/novel.507/ENSG00000168032/ENSG00000101412/ENSG00000171791/ENSG00000145386/ENSG00000111335/ENSG00000137462 | VIM/-/ENTPD3/E2F1/BCL2/CCNA2/OAS2/TLR2 | hsa:7431/hsa:1870/hsa:956/hsa:1869/hsa:596/hsa:890/hsa:4939/hsa:7097 | 8 | 5 | ENSG00000026025/novel.507/ENSG00000101412/ENSG00000171791/ENSG00000145386 | 3 | ENSG00000168032/ENSG00000111335/ENSG00000137462 |
| hsa04934 | Cushing syndrome | 6\|253 | 166\|9228 | 0.3039805 | 0.9084473 | novel.507/ENSG00000157613/ENSG00000108551/ENSG00000101412/ENSG00000164742/novel.600 | -/CREB3L1/RASD1/E2F1/ADCY1/- | hsa:1870/hsa:90993/hsa:51655/hsa:1869/hsa:107/hsa:1388 | 6 | 4 | novel.507/ENSG00000101412/ENSG00000164742/novel.600 | 2 | ENSG00000157613/ENSG00000108551 |
| hsa00010 | Glycolysis / Gluconeogenesis | 4\|253 | 104\|9228 | 0.3187916 | 0.9156866 | ENSG00000259583/ENSG00000184254/ENSG00000137124/ENSG00000186197 | AC015712.2/ALDH1A3/ALDH1B1/EDARADD | hsa:220/hsa:220/hsa:219/hsa:2023 | 4 | 1 | ENSG00000137124 | 3 | ENSG00000259583/ENSG00000184254/ENSG00000186197 |
| hsa00860 | Porphyrin and chlorophyll metabolism | 2\|253 | 43\|9228 | 0.3307556 | 0.9156866 | ENSG00000047457/ENSG00000240216 | CP/CPHL1P | hsa:1356/hsa:1356 | 2 | 0 |  | 2 | ENSG00000047457/ENSG00000240216 |
| hsa05410 | Hypertrophic cardiomyopathy (HCM) | 4\|253 | 106\|9228 | 0.3312829 | 0.9156866 | ENSG00000078401/ENSG00000005961/ENSG00000092969/ENSG00000115221 | EDN1/ITGA2B/TGFB2/ITGB6 | hsa:1906/hsa:3674/hsa:7042/hsa:3694 | 4 | 1 | ENSG00000005961 | 3 | ENSG00000078401/ENSG00000092969/ENSG00000115221 |
| hsa05034 | Alcoholism | 7\|253 | 205\|9228 | 0.3313760 | 0.9156866 | ENSG00000157613/ENSG00000127588/ENSG00000184260/ENSG00000124635/ENSG00000180176/novel.600/ENSG00000218690 | CREB3L1/GNG13/HIST2H2AC/HIST1H2BJ/TH/-/HIST1H2APS4 | hsa:90993/hsa:51764/hsa:8338/hsa:8970/hsa:7054/hsa:1388/hsa:8336 | 7 | 6 | ENSG00000127588/ENSG00000184260/ENSG00000124635/ENSG00000180176/novel.600/ENSG00000218690 | 1 | ENSG00000157613 |
| hsa04015 | Rap1 signaling pathway | 8\|253 | 240\|9228 | 0.3372041 | 0.9156866 | ENSG00000005961/ENSG00000160867/ENSG00000152689/ENSG00000128052/ENSG00000164742/ENSG00000082074/ENSG00000037280/ENSG00000233328 | ITGA2B/FGFR4/RASGRP3/KDR/ADCY1/FYB1/FLT4/PFN1P1 | hsa:3674/hsa:2264/hsa:25780/hsa:3791/hsa:107/hsa:2533/hsa:2324/hsa:5216 | 8 | 5 | ENSG00000005961/ENSG00000160867/ENSG00000164742/ENSG00000037280/ENSG00000233328 | 3 | ENSG00000152689/ENSG00000128052/ENSG00000082074 |
| hsa04022 | cGMP-PKG signaling pathway | 7\|253 | 207\|9228 | 0.3402989 | 0.9156866 | ENSG00000185532/ENSG00000157613/ENSG00000116741/ENSG00000164742/ENSG00000168398/novel.600/ENSG00000121361 | PRKG1/CREB3L1/RGS2/ADCY1/BDKRB2/-/KCNJ8 | hsa:5592/hsa:90993/hsa:5997/hsa:107/hsa:624/hsa:1388/hsa:3764 | 7 | 3 | ENSG00000164742/novel.600/ENSG00000121361 | 4 | ENSG00000185532/ENSG00000157613/ENSG00000116741/ENSG00000168398 |
| hsa05210 | Colorectal cancer | 4\|253 | 108\|9228 | 0.3438001 | 0.9156866 | ENSG00000092969/ENSG00000109321/ENSG00000089685/ENSG00000171791 | TGFB2/AREG/BIRC5/BCL2 | hsa:7042/hsa:374/hsa:332/hsa:596 | 4 | 2 | ENSG00000089685/ENSG00000171791 | 2 | ENSG00000092969/ENSG00000109321 |
| hsa04060 | Cytokine-cytokine receptor interaction | 7\|253 | 208\|9228 | 0.3447726 | 0.9156866 | ENSG00000107562/ENSG00000181634/ENSG00000169429/ENSG00000092969/ENSG00000006606/ENSG00000081041/ENSG00000104998 | CXCL12/TNFSF15/CXCL8/TGFB2/CCL26/CXCL2/IL27RA | hsa:6387/hsa:9966/hsa:3576/hsa:7042/hsa:10344/hsa:2920/hsa:9466 | 7 | 2 | ENSG00000107562/ENSG00000104998 | 5 | ENSG00000181634/ENSG00000169429/ENSG00000092969/ENSG00000006606/ENSG00000081041 |
| hsa04062 | Chemokine signaling pathway | 6\|253 | 175\|9228 | 0.3477240 | 0.9156866 | ENSG00000107562/ENSG00000169429/ENSG00000127588/ENSG00000164742/ENSG00000006606/ENSG00000081041 | CXCL12/CXCL8/GNG13/ADCY1/CCL26/CXCL2 | hsa:6387/hsa:3576/hsa:51764/hsa:107/hsa:10344/hsa:2920 | 6 | 3 | ENSG00000107562/ENSG00000127588/ENSG00000164742 | 3 | ENSG00000169429/ENSG00000006606/ENSG00000081041 |
| hsa04917 | Prolactin signaling pathway | 3\|253 | 77\|9228 | 0.3538560 | 0.9156866 | ENSG00000135346/ENSG00000114737/ENSG00000180176 | CGA/CISH/TH | hsa:1081/hsa:1154/hsa:7054 | 3 | 2 | ENSG00000114737/ENSG00000180176 | 1 | ENSG00000135346 |
| hsa00603 | Glycosphingolipid biosynthesis - globo and isoglobo series | 1\|253 | 16\|9228 | 0.3592766 | 0.9156866 | ENSG00000102393 | GLA | hsa:2717 | 1 | 1 | ENSG00000102393 | 0 |  |
| hsa04970 | Salivary secretion | 3\|253 | 78\|9228 | 0.3613850 | 0.9156866 | ENSG00000185532/ENSG00000104783/ENSG00000164742 | PRKG1/KCNN4/ADCY1 | hsa:5592/hsa:3783/hsa:107 | 3 | 2 | ENSG00000104783/ENSG00000164742 | 1 | ENSG00000185532 |
| hsa05414 | Dilated cardiomyopathy (DCM) | 4\|253 | 112\|9228 | 0.3688581 | 0.9156866 | ENSG00000005961/ENSG00000092969/ENSG00000164742/ENSG00000115221 | ITGA2B/TGFB2/ADCY1/ITGB6 | hsa:3674/hsa:7042/hsa:107/hsa:3694 | 4 | 2 | ENSG00000005961/ENSG00000164742 | 2 | ENSG00000092969/ENSG00000115221 |
| hsa05140 | Leishmaniasis | 3\|253 | 79\|9228 | 0.3689009 | 0.9156866 | ENSG00000092969/ENSG00000116701/ENSG00000137462 | TGFB2/NCF2/TLR2 | hsa:7042/hsa:4688/hsa:7097 | 3 | 0 |  | 3 | ENSG00000092969/ENSG00000116701/ENSG00000137462 |
| hsa04962 | Vasopressin-regulated water reabsorption | 2\|253 | 47\|9228 | 0.3707810 | 0.9156866 | ENSG00000157613/ENSG00000111348 | CREB3L1/ARHGDIB | hsa:90993/hsa:397 | 2 | 0 |  | 2 | ENSG00000157613/ENSG00000111348 |
| hsa04979 | Cholesterol metabolism | 2\|253 | 47\|9228 | 0.3707810 | 0.9156866 | ENSG00000101670/ENSG00000167780 | LIPG/SOAT2 | hsa:9388/hsa:8435 | 2 | 1 | ENSG00000167780 | 1 | ENSG00000101670 |
| hsa00604 | Glycosphingolipid biosynthesis - ganglio series | 1\|253 | 17\|9228 | 0.3768736 | 0.9156866 | ENSG00000135454 | B4GALNT1 | hsa:2583 | 1 | 1 | ENSG00000135454 | 0 |  |
| hsa03450 | Non-homologous end-joining | 1\|253 | 17\|9228 | 0.3768736 | 0.9156866 | ENSG00000168496 | FEN1 | hsa:2237 | 1 | 1 | ENSG00000168496 | 0 |  |
| hsa05224 | Breast cancer | 5\|253 | 147\|9228 | 0.3771403 | 0.9156866 | ENSG00000082175/ENSG00000135547/novel.507/ENSG00000101412/ENSG00000037280 | PGR/HEY2/-/E2F1/FLT4 | hsa:5241/hsa:23493/hsa:1870/hsa:1869/hsa:2324 | 5 | 5 | ENSG00000082175/ENSG00000135547/novel.507/ENSG00000101412/ENSG00000037280 | 0 |  |
| hsa05226 | Gastric cancer | 5\|253 | 148\|9228 | 0.3826143 | 0.9156866 | novel.507/ENSG00000092969/ENSG00000101412/ENSG00000171791/ENSG00000164362 | -/TGFB2/E2F1/BCL2/TERT | hsa:1870/hsa:7042/hsa:1869/hsa:596/hsa:7015 | 5 | 4 | novel.507/ENSG00000101412/ENSG00000171791/ENSG00000164362 | 1 | ENSG00000092969 |
| hsa05163 | Human cytomegalovirus infection | 8\|253 | 251\|9228 | 0.3830724 | 0.9156866 | ENSG00000107562/novel.507/ENSG00000157613/ENSG00000169429/ENSG00000127588/ENSG00000101412/ENSG00000164742/novel.600 | CXCL12/-/CREB3L1/CXCL8/GNG13/E2F1/ADCY1/- | hsa:6387/hsa:1870/hsa:90993/hsa:3576/hsa:51764/hsa:1869/hsa:107/hsa:1388 | 8 | 6 | ENSG00000107562/novel.507/ENSG00000127588/ENSG00000101412/ENSG00000164742/novel.600 | 2 | ENSG00000157613/ENSG00000169429 |
| hsa03320 | PPAR signaling pathway | 3\|253 | 81\|9228 | 0.3838840 | 0.9156866 | ENSG00000214456/ENSG00000134240/ENSG00000132170 | PLIN5/HMGCS2/PPARG | hsa:440503/hsa:3158/hsa:5468 | 3 | 1 | ENSG00000214456 | 2 | ENSG00000134240/ENSG00000132170 |
| hsa05215 | Prostate cancer | 4\|253 | 115\|9228 | 0.3876217 | 0.9161967 | novel.507/ENSG00000157613/ENSG00000101412/ENSG00000171791 | -/CREB3L1/E2F1/BCL2 | hsa:1870/hsa:90993/hsa:1869/hsa:596 | 4 | 3 | novel.507/ENSG00000101412/ENSG00000171791 | 1 | ENSG00000157613 |
| hsa05033 | Nicotine addiction | 1\|253 | 18\|9228 | 0.3939891 | 0.9196091 | ENSG00000187730 | GABRD | hsa:2563 | 1 | 1 | ENSG00000187730 | 0 |  |
| hsa05167 | Kaposi sarcoma-associated herpesvirus infection | 7\|253 | 220\|9228 | 0.3988414 | 0.9196091 | novel.507/ENSG00000169429/ENSG00000127588/ENSG00000159200/ENSG00000101412/ENSG00000139112/ENSG00000081041 | -/CXCL8/GNG13/RCAN1/E2F1/GABARAPL1/CXCL2 | hsa:1870/hsa:3576/hsa:51764/hsa:1827/hsa:1869/hsa:23710/hsa:2920 | 7 | 3 | novel.507/ENSG00000127588/ENSG00000101412 | 4 | ENSG00000169429/ENSG00000159200/ENSG00000139112/ENSG00000081041 |
| hsa01230 | Biosynthesis of amino acids | 4\|253 | 118\|9228 | 0.4063140 | 0.9196091 | ENSG00000104524/ENSG00000186197/ENSG00000151224/ENSG00000092621 | PYCR3/EDARADD/MAT1A/PHGDH | hsa:65263/hsa:2023/hsa:4143/hsa:26227 | 4 | 3 | ENSG00000104524/ENSG00000151224/ENSG00000092621 | 1 | ENSG00000186197 |
| hsa04913 | Ovarian steroidogenesis | 2\|253 | 51\|9228 | 0.4098283 | 0.9196091 | ENSG00000135346/ENSG00000164742 | CGA/ADCY1 | hsa:1081/hsa:107 | 2 | 1 | ENSG00000164742 | 1 | ENSG00000135346 |
| hsa04614 | Renin-angiotensin system | 1\|253 | 19\|9228 | 0.4106363 | 0.9196091 | ENSG00000166825 | ANPEP | hsa:290 | 1 | 1 | ENSG00000166825 | 0 |  |
| hsa00310 | Lysine degradation | 3\|253 | 85\|9228 | 0.4135894 | 0.9196091 | ENSG00000008311/ENSG00000101945/ENSG00000137124 | AASS/SUV39H1/ALDH1B1 | hsa:10157/hsa:6839/hsa:219 | 3 | 2 | ENSG00000101945/ENSG00000137124 | 1 | ENSG00000008311 |
| hsa04120 | Ubiquitin mediated proteolysis | 7\|253 | 224\|9228 | 0.4169108 | 0.9196091 | ENSG00000224126/ENSG00000117399/ENSG00000108106/ENSG00000114423/ENSG00000101871/ENSG00000023445/ENSG00000224080 | UBE2SP2/CDC20/UBE2S/CBLB/MID1/BIRC3/UBE2FP1 | hsa:27338/hsa:991/hsa:27338/hsa:868/hsa:4281/hsa:330/hsa:140739 | 7 | 4 | ENSG00000224126/ENSG00000117399/ENSG00000108106/ENSG00000224080 | 3 | ENSG00000114423/ENSG00000101871/ENSG00000023445 |
| hsa00910 | Nitrogen metabolism | 1\|253 | 20\|9228 | 0.4268280 | 0.9196091 | ENSG00000074410 | CA12 | hsa:771 | 1 | 1 | ENSG00000074410 | 0 |  |
| hsa04935 | Growth hormone synthesis, secretion and action | 4\|253 | 122\|9228 | 0.4310606 | 0.9196091 | ENSG00000157613/ENSG00000146674/ENSG00000164742/novel.600 | CREB3L1/IGFBP3/ADCY1/- | hsa:90993/hsa:3486/hsa:107/hsa:1388 | 4 | 2 | ENSG00000164742/novel.600 | 2 | ENSG00000157613/ENSG00000146674 |
| hsa04145 | Phagosome | 6\|253 | 192\|9228 | 0.4313238 | 0.9196091 | ENSG00000214222/ENSG00000123416/ENSG00000116701/ENSG00000102879/ENSG00000137462/ENSG00000104833 | TUBBP2/TUBA1B/NCF2/CORO1A/TLR2/TUBB4A | hsa:203068/hsa:10376/hsa:4688/hsa:11151/hsa:7097/hsa:10382 | 6 | 4 | ENSG00000214222/ENSG00000123416/ENSG00000102879/ENSG00000104833 | 2 | ENSG00000116701/ENSG00000137462 |
| hsa04922 | Glucagon signaling pathway | 4\|253 | 123\|9228 | 0.4372069 | 0.9196091 | ENSG00000157613/ENSG00000109819/ENSG00000275993/ENSG00000117394 | CREB3L1/PPARGC1A/SIK1B/SLC2A1 | hsa:90993/hsa:10891/hsa:102724428/hsa:6513 | 4 | 1 | ENSG00000117394 | 3 | ENSG00000157613/ENSG00000109819/ENSG00000275993 |
| hsa00260 | Glycine, serine and threonine metabolism | 2\|253 | 54\|9228 | 0.4383267 | 0.9196091 | ENSG00000123453/ENSG00000092621 | SARDH/PHGDH | hsa:1757/hsa:26227 | 2 | 2 | ENSG00000123453/ENSG00000092621 | 0 |  |
| hsa00053 | Ascorbate and aldarate metabolism | 1\|253 | 21\|9228 | 0.4425765 | 0.9196091 | ENSG00000137124 | ALDH1B1 | hsa:219 | 1 | 1 | ENSG00000137124 | 0 |  |
| hsa05211 | Renal cell carcinoma | 3\|253 | 90\|9228 | 0.4500597 | 0.9196091 | ENSG00000116016/ENSG00000092969/ENSG00000117394 | EPAS1/TGFB2/SLC2A1 | hsa:2034/hsa:7042/hsa:6513 | 3 | 1 | ENSG00000117394 | 2 | ENSG00000116016/ENSG00000092969 |
| hsa04371 | Apelin signaling pathway | 5\|253 | 161\|9228 | 0.4532691 | 0.9196091 | ENSG00000127588/ENSG00000118523/ENSG00000109819/ENSG00000139112/ENSG00000164742 | GNG13/CTGF/PPARGC1A/GABARAPL1/ADCY1 | hsa:51764/hsa:1490/hsa:10891/hsa:23710/hsa:107 | 5 | 2 | ENSG00000127588/ENSG00000164742 | 3 | ENSG00000118523/ENSG00000109819/ENSG00000139112 |
| hsa04961 | Endocrine and other factor-regulated calcium reabsorption | 2\|253 | 56\|9228 | 0.4569062 | 0.9196091 | ENSG00000197959/ENSG00000168398 | DNM3/BDKRB2 | hsa:26052/hsa:624 | 2 | 0 |  | 2 | ENSG00000197959/ENSG00000168398 |
| hsa04012 | ErbB signaling pathway | 3\|253 | 94\|9228 | 0.4785647 | 0.9196091 | ENSG00000109321/ENSG00000174808/ENSG00000114423 | AREG/BTC/CBLB | hsa:374/hsa:685/hsa:868 | 3 | 0 |  | 3 | ENSG00000109321/ENSG00000174808/ENSG00000114423 |
| hsa04657 | IL-17 signaling pathway | 3\|253 | 94\|9228 | 0.4785647 | 0.9196091 | ENSG00000169429/ENSG00000137745/ENSG00000081041 | CXCL8/MMP13/CXCL2 | hsa:3576/hsa:4322/hsa:2920 | 3 | 0 |  | 3 | ENSG00000169429/ENSG00000137745/ENSG00000081041 |
| hsa04972 | Pancreatic secretion | 3\|253 | 94\|9228 | 0.4785647 | 0.9196091 | ENSG00000080493/ENSG00000164742/ENSG00000188257 | SLC4A4/ADCY1/PLA2G2A | hsa:8671/hsa:107/hsa:5320 | 3 | 1 | ENSG00000164742 | 2 | ENSG00000080493/ENSG00000188257 |
| hsa00280 | Valine, leucine and isoleucine degradation | 2\|253 | 59\|9228 | 0.4840994 | 0.9196091 | ENSG00000134240/ENSG00000137124 | HMGCS2/ALDH1B1 | hsa:3158/hsa:219 | 2 | 1 | ENSG00000137124 | 1 | ENSG00000134240 |
| hsa05212 | Pancreatic cancer | 3\|253 | 96\|9228 | 0.4925592 | 0.9196091 | novel.507/ENSG00000092969/ENSG00000101412 | -/TGFB2/E2F1 | hsa:1870/hsa:7042/hsa:1869 | 3 | 2 | novel.507/ENSG00000101412 | 1 | ENSG00000092969 |
| hsa05225 | Hepatocellular carcinoma | 6\|253 | 205\|9228 | 0.4942001 | 0.9196091 | novel.507/ENSG00000213366/ENSG00000092969/ENSG00000101412/ENSG00000164362/ENSG00000241720 | -/GSTM2/TGFB2/E2F1/TERT/AL158847.1 | hsa:1870/hsa:2946/hsa:7042/hsa:1869/hsa:7015/hsa:2947 | 6 | 3 | novel.507/ENSG00000101412/ENSG00000164362 | 3 | ENSG00000213366/ENSG00000092969/ENSG00000241720 |
| hsa04728 | Dopaminergic synapse | 4\|253 | 133\|9228 | 0.4974588 | 0.9196091 | ENSG00000157613/ENSG00000127588/ENSG00000180176/novel.600 | CREB3L1/GNG13/TH/- | hsa:90993/hsa:51764/hsa:7054/hsa:1388 | 4 | 3 | ENSG00000127588/ENSG00000180176/novel.600 | 1 | ENSG00000157613 |
| hsa04152 | AMPK signaling pathway | 4\|253 | 134\|9228 | 0.5033397 | 0.9196091 | ENSG00000157613/ENSG00000109819/ENSG00000145386/ENSG00000132170 | CREB3L1/PPARGC1A/CCNA2/PPARG | hsa:90993/hsa:10891/hsa:890/hsa:5468 | 4 | 1 | ENSG00000145386 | 3 | ENSG00000157613/ENSG00000109819/ENSG00000132170 |
| hsa00534 | Glycosaminoglycan biosynthesis - heparan sulfate / heparin | 1\|253 | 26\|9228 | 0.5150838 | 0.9196091 | ENSG00000002587 | HS3ST1 | hsa:9957 | 1 | 0 |  | 1 | ENSG00000002587 |
| hsa05320 | Autoimmune thyroid disease | 1\|253 | 26\|9228 | 0.5150838 | 0.9196091 | ENSG00000135346 | CGA | hsa:1081 | 1 | 0 |  | 1 | ENSG00000135346 |
| hsa05220 | Chronic myeloid leukemia | 3\|253 | 100\|9228 | 0.5199796 | 0.9196091 | novel.507/ENSG00000092969/ENSG00000101412 | -/TGFB2/E2F1 | hsa:1870/hsa:7042/hsa:1869 | 3 | 2 | novel.507/ENSG00000101412 | 1 | ENSG00000092969 |
| hsa00592 | alpha-Linolenic acid metabolism | 1\|253 | 27\|9228 | 0.5284161 | 0.9196091 | ENSG00000188257 | PLA2G2A | hsa:5320 | 1 | 0 |  | 1 | ENSG00000188257 |
| hsa04916 | Melanogenesis | 3\|253 | 102\|9228 | 0.5333874 | 0.9196091 | ENSG00000078401/ENSG00000157613/ENSG00000164742 | EDN1/CREB3L1/ADCY1 | hsa:1906/hsa:90993/hsa:107 | 3 | 1 | ENSG00000164742 | 2 | ENSG00000078401/ENSG00000157613 |
| hsa04925 | Aldosterone synthesis and secretion | 3\|253 | 103\|9228 | 0.5400122 | 0.9196091 | ENSG00000157613/ENSG00000164742/novel.600 | CREB3L1/ADCY1/- | hsa:90993/hsa:107/hsa:1388 | 3 | 2 | ENSG00000164742/novel.600 | 1 | ENSG00000157613 |
| hsa00670 | One carbon pool by folate | 1\|253 | 28\|9228 | 0.5413833 | 0.9196091 | ENSG00000176890 | TYMS | hsa:7298 | 1 | 1 | ENSG00000176890 | 0 |  |
| hsa00790 | Folate biosynthesis | 1\|253 | 28\|9228 | 0.5413833 | 0.9196091 | ENSG00000180176 | TH | hsa:7054 | 1 | 1 | ENSG00000180176 | 0 |  |
| hsa04964 | Proximal tubule bicarbonate reclamation | 1\|253 | 28\|9228 | 0.5413833 | 0.9196091 | ENSG00000080493 | SLC4A4 | hsa:8671 | 1 | 0 |  | 1 | ENSG00000080493 |
| hsa05152 | Tuberculosis | 5\|253 | 178\|9228 | 0.5419428 | 0.9196091 | ENSG00000092969/ENSG00000171791/ENSG00000102879/ENSG00000137462/ENSG00000187796 | TGFB2/BCL2/CORO1A/TLR2/CARD9 | hsa:7042/hsa:596/hsa:11151/hsa:7097/hsa:64170 | 5 | 3 | ENSG00000171791/ENSG00000102879/ENSG00000187796 | 2 | ENSG00000092969/ENSG00000137462 |
| hsa04611 | Platelet activation | 4\|253 | 141\|9228 | 0.5436196 | 0.9196091 | ENSG00000185532/ENSG00000005961/ENSG00000164742/ENSG00000168542 | PRKG1/ITGA2B/ADCY1/COL3A1 | hsa:5592/hsa:3674/hsa:107/hsa:1281 | 4 | 3 | ENSG00000005961/ENSG00000164742/ENSG00000168542 | 1 | ENSG00000185532 |
| hsa04640 | Hematopoietic cell lineage | 2\|253 | 67\|9228 | 0.5523359 | 0.9196091 | ENSG00000005961/ENSG00000166825 | ITGA2B/ANPEP | hsa:3674/hsa:290 | 2 | 2 | ENSG00000005961/ENSG00000166825 | 0 |  |
| hsa03018 | RNA degradation | 3\|253 | 105\|9228 | 0.5530993 | 0.9196091 | ENSG00000159388/ENSG00000130520/ENSG00000186197 | BTG2/LSM4/EDARADD | hsa:7832/hsa:25804/hsa:2023 | 3 | 1 | ENSG00000130520 | 2 | ENSG00000159388/ENSG00000186197 |
| hsa00052 | Galactose metabolism | 1\|253 | 29\|9228 | 0.5539952 | 0.9196091 | ENSG00000102393 | GLA | hsa:2717 | 1 | 1 | ENSG00000102393 | 0 |  |
| hsa04672 | Intestinal immune network for IgA production | 1\|253 | 29\|9228 | 0.5539952 | 0.9196091 | ENSG00000107562 | CXCL12 | hsa:6387 | 1 | 1 | ENSG00000107562 | 0 |  |
| hsa04724 | Glutamatergic synapse | 3\|253 | 106\|9228 | 0.5595599 | 0.9196091 | ENSG00000127588/ENSG00000164742/ENSG00000164418 | GNG13/ADCY1/GRIK2 | hsa:51764/hsa:107/hsa:2898 | 3 | 2 | ENSG00000127588/ENSG00000164742 | 1 | ENSG00000164418 |
| hsa05162 | Measles | 4\|253 | 144\|9228 | 0.5603662 | 0.9196091 | ENSG00000171791/ENSG00000114423/ENSG00000111335/ENSG00000137462 | BCL2/CBLB/OAS2/TLR2 | hsa:596/hsa:868/hsa:4939/hsa:7097 | 4 | 1 | ENSG00000171791 | 3 | ENSG00000114423/ENSG00000111335/ENSG00000137462 |
| hsa04810 | Regulation of actin cytoskeleton | 7\|253 | 257\|9228 | 0.5614472 | 0.9196091 | ENSG00000107562/ENSG00000005961/ENSG00000160867/ENSG00000168539/ENSG00000168398/ENSG00000115221/ENSG00000233328 | CXCL12/ITGA2B/FGFR4/CHRM1/BDKRB2/ITGB6/PFN1P1 | hsa:6387/hsa:3674/hsa:2264/hsa:1128/hsa:624/hsa:3694/hsa:5216 | 7 | 5 | ENSG00000107562/ENSG00000005961/ENSG00000160867/ENSG00000168539/ENSG00000233328 | 2 | ENSG00000168398/ENSG00000115221 |
| hsa00564 | Glycerophospholipid metabolism | 3\|253 | 107\|9228 | 0.5659644 | 0.9196091 | ENSG00000143845/ENSG00000188257/ENSG00000087085 | ETNK2/PLA2G2A/ACHE | hsa:55224/hsa:5320/hsa:43 | 3 | 1 | ENSG00000143845 | 2 | ENSG00000188257/ENSG00000087085 |
| hsa05218 | Melanoma | 2\|253 | 70\|9228 | 0.5762392 | 0.9196091 | novel.507/ENSG00000101412 | -/E2F1 | hsa:1870/hsa:1869 | 2 | 2 | novel.507/ENSG00000101412 | 0 |  |
| hsa04210 | Apoptosis | 5\|253 | 185\|9228 | 0.5764952 | 0.9196091 | ENSG00000162909/ENSG00000123416/ENSG00000089685/ENSG00000171791/ENSG00000023445 | CAPN2/TUBA1B/BIRC5/BCL2/BIRC3 | hsa:824/hsa:10376/hsa:332/hsa:596/hsa:330 | 5 | 3 | ENSG00000123416/ENSG00000089685/ENSG00000171791 | 2 | ENSG00000162909/ENSG00000023445 |
| hsa04217 | Necroptosis | 6\|253 | 223\|9228 | 0.5770030 | 0.9196091 | ENSG00000162909/ENSG00000138378/ENSG00000184260/ENSG00000171791/ENSG00000023445/ENSG00000218690 | CAPN2/STAT4/HIST2H2AC/BCL2/BIRC3/HIST1H2APS4 | hsa:824/hsa:6775/hsa:8338/hsa:596/hsa:330/hsa:8336 | 6 | 3 | ENSG00000184260/ENSG00000171791/ENSG00000218690 | 3 | ENSG00000162909/ENSG00000138378/ENSG00000023445 |
| hsa00500 | Starch and sucrose metabolism | 1\|253 | 31\|9228 | 0.5781921 | 0.9196091 | ENSG00000165434 | PGM2L1 | hsa:283209 | 1 | 0 |  | 1 | ENSG00000165434 |
| hsa00591 | Linoleic acid metabolism | 1\|253 | 31\|9228 | 0.5781921 | 0.9196091 | ENSG00000188257 | PLA2G2A | hsa:5320 | 1 | 0 |  | 1 | ENSG00000188257 |
| hsa00900 | Terpenoid backbone biosynthesis | 1\|253 | 31\|9228 | 0.5781921 | 0.9196091 | ENSG00000134240 | HMGCS2 | hsa:3158 | 1 | 0 |  | 1 | ENSG00000134240 |
| hsa04630 | JAK-STAT signaling pathway | 4\|253 | 148\|9228 | 0.5821711 | 0.9196091 | ENSG00000114737/ENSG00000138378/ENSG00000171791/ENSG00000104998 | CISH/STAT4/BCL2/IL27RA | hsa:1154/hsa:6775/hsa:596/hsa:9466 | 4 | 3 | ENSG00000114737/ENSG00000171791/ENSG00000104998 | 1 | ENSG00000138378 |
| hsa00590 | Arachidonic acid metabolism | 2\|253 | 71\|9228 | 0.5839980 | 0.9196091 | ENSG00000256612/ENSG00000188257 | CYP2B7P/PLA2G2A | hsa:1555/hsa:5320 | 2 | 0 |  | 2 | ENSG00000256612/ENSG00000188257 |
| hsa04920 | Adipocytokine signaling pathway | 2\|253 | 71\|9228 | 0.5839980 | 0.9196091 | ENSG00000109819/ENSG00000117394 | PPARGC1A/SLC2A1 | hsa:10891/hsa:6513 | 2 | 1 | ENSG00000117394 | 1 | ENSG00000109819 |
| hsa04625 | C-type lectin receptor signaling pathway | 3\|253 | 111\|9228 | 0.5910072 | 0.9196091 | ENSG00000179388/ENSG00000114423/ENSG00000187796 | EGR3/CBLB/CARD9 | hsa:1960/hsa:868/hsa:64170 | 3 | 2 | ENSG00000179388/ENSG00000187796 | 1 | ENSG00000114423 |
| hsa05221 | Acute myeloid leukemia | 2\|253 | 72\|9228 | 0.5916520 | 0.9196091 | ENSG00000131759/ENSG00000145386 | RARA/CCNA2 | hsa:5914/hsa:890 | 2 | 2 | ENSG00000131759/ENSG00000145386 | 0 |  |
| hsa00650 | Butanoate metabolism | 1\|253 | 33\|9228 | 0.6010811 | 0.9196091 | ENSG00000134240 | HMGCS2 | hsa:3158 | 1 | 0 |  | 1 | ENSG00000134240 |
| hsa01040 | Biosynthesis of unsaturated fatty acids | 1\|253 | 33\|9228 | 0.6010811 | 0.9196091 | ENSG00000097021 | ACOT7 | hsa:11332 | 1 | 1 | ENSG00000097021 | 0 |  |
| hsa04066 | HIF-1 signaling pathway | 4\|253 | 152\|9228 | 0.6033465 | 0.9196091 | ENSG00000078401/ENSG00000171791/ENSG00000117394/ENSG00000186197 | EDN1/BCL2/SLC2A1/EDARADD | hsa:1906/hsa:596/hsa:6513/hsa:2023 | 4 | 2 | ENSG00000171791/ENSG00000117394 | 2 | ENSG00000078401/ENSG00000186197 |
| hsa03460 | Fanconi anemia pathway | 2\|253 | 74\|9228 | 0.6066450 | 0.9196091 | ENSG00000154920/ENSG00000221829 | EME1/FANCG | hsa:146956/hsa:2189 | 2 | 2 | ENSG00000154920/ENSG00000221829 | 0 |  |
| hsa04014 | Ras signaling pathway | 7\|253 | 269\|9228 | 0.6100694 | 0.9196091 | ENSG00000160867/ENSG00000152689/ENSG00000128052/ENSG00000127588/ENSG00000037280/ENSG00000143344/ENSG00000188257 | FGFR4/RASGRP3/KDR/GNG13/FLT4/RGL1/PLA2G2A | hsa:2264/hsa:25780/hsa:3791/hsa:51764/hsa:2324/hsa:23179/hsa:5320 | 7 | 3 | ENSG00000160867/ENSG00000127588/ENSG00000037280 | 4 | ENSG00000152689/ENSG00000128052/ENSG00000143344/ENSG00000188257 |
| hsa04390 | Hippo signaling pathway | 5\|253 | 193\|9228 | 0.6142526 | 0.9196091 | ENSG00000118523/ENSG00000092969/ENSG00000109321/ENSG00000089685/ENSG00000023445 | CTGF/TGFB2/AREG/BIRC5/BIRC3 | hsa:1490/hsa:7042/hsa:374/hsa:332/hsa:330 | 5 | 1 | ENSG00000089685 | 4 | ENSG00000118523/ENSG00000092969/ENSG00000109321/ENSG00000023445 |
| hsa04261 | Adrenergic signaling in cardiomyocytes | 4\|253 | 155\|9228 | 0.6187982 | 0.9196091 | ENSG00000157613/ENSG00000171791/ENSG00000164742/novel.600 | CREB3L1/BCL2/ADCY1/- | hsa:90993/hsa:596/hsa:107/hsa:1388 | 4 | 3 | ENSG00000171791/ENSG00000164742/novel.600 | 1 | ENSG00000157613 |
| hsa04380 | Osteoclast differentiation | 3\|253 | 116\|9228 | 0.6209788 | 0.9196091 | ENSG00000092969/ENSG00000116701/ENSG00000132170 | TGFB2/NCF2/PPARG | hsa:7042/hsa:4688/hsa:5468 | 3 | 0 |  | 3 | ENSG00000092969/ENSG00000116701/ENSG00000132170 |
| hsa04931 | Insulin resistance | 3\|253 | 116\|9228 | 0.6209788 | 0.9196091 | ENSG00000157613/ENSG00000109819/ENSG00000117394 | CREB3L1/PPARGC1A/SLC2A1 | hsa:90993/hsa:10891/hsa:6513 | 3 | 1 | ENSG00000117394 | 2 | ENSG00000157613/ENSG00000109819 |
| hsa04662 | B cell receptor signaling pathway | 2\|253 | 76\|9228 | 0.6212183 | 0.9196091 | ENSG00000152689/ENSG00000070190 | RASGRP3/DAPP1 | hsa:25780/hsa:27071 | 2 | 0 |  | 2 | ENSG00000152689/ENSG00000070190 |
| hsa00512 | Mucin type O-glycan biosynthesis | 1\|253 | 35\|9228 | 0.6227327 | 0.9196091 | ENSG00000144278 | GALNT13 | hsa:114805 | 1 | 0 |  | 1 | ENSG00000144278 |
| hsa05134 | Legionellosis | 3\|253 | 117\|9228 | 0.6267915 | 0.9196091 | ENSG00000169429/ENSG00000081041/ENSG00000137462 | CXCL8/CXCL2/TLR2 | hsa:3576/hsa:2920/hsa:7097 | 3 | 0 |  | 3 | ENSG00000169429/ENSG00000081041/ENSG00000137462 |
| hsa05223 | Non-small cell lung cancer | 2\|253 | 77\|9228 | 0.6283479 | 0.9196091 | novel.507/ENSG00000101412 | -/E2F1 | hsa:1870/hsa:1869 | 2 | 2 | novel.507/ENSG00000101412 | 0 |  |
| hsa00062 | Fatty acid elongation | 1\|253 | 36\|9228 | 0.6331155 | 0.9196091 | ENSG00000097021 | ACOT7 | hsa:11332 | 1 | 1 | ENSG00000097021 | 0 |  |
| hsa03020 | RNA polymerase | 1\|253 | 36\|9228 | 0.6331155 | 0.9196091 | ENSG00000105258 | POLR2I | hsa:5438 | 1 | 1 | ENSG00000105258 | 0 |  |
| hsa05020 | Prion diseases | 1\|253 | 38\|9228 | 0.6530348 | 0.9413801 | ENSG00000135862 | LAMC1 | hsa:3915 | 1 | 0 |  | 1 | ENSG00000135862 |
| hsa04740 | Olfactory transduction | 3\|253 | 123\|9228 | 0.6603771 | 0.9413801 | ENSG00000185532/ENSG00000127588/ENSG00000116741 | PRKG1/GNG13/RGS2 | hsa:5592/hsa:51764/hsa:5997 | 3 | 1 | ENSG00000127588 | 2 | ENSG00000185532/ENSG00000116741 |
| hsa05322 | Systemic lupus erythematosus | 3\|253 | 123\|9228 | 0.6603771 | 0.9413801 | ENSG00000184260/ENSG00000124635/ENSG00000218690 | HIST2H2AC/HIST1H2BJ/HIST1H2APS4 | hsa:8338/hsa:8970/hsa:8336 | 3 | 3 | ENSG00000184260/ENSG00000124635/ENSG00000218690 | 0 |  |
| hsa00051 | Fructose and mannose metabolism | 1\|253 | 39\|9228 | 0.6625867 | 0.9413801 | ENSG00000138030 | KHK | hsa:3795 | 1 | 1 | ENSG00000138030 | 0 |  |
| hsa04068 | FoxO signaling pathway | 4\|253 | 165\|9228 | 0.6675256 | 0.9432427 | ENSG00000166851/ENSG00000092969/ENSG00000139112/ENSG00000157456 | PLK1/TGFB2/GABARAPL1/CCNB2 | hsa:5347/hsa:7042/hsa:23710/hsa:9133 | 4 | 2 | ENSG00000166851/ENSG00000157456 | 2 | ENSG00000092969/ENSG00000139112 |
| hsa04975 | Fat digestion and absorption | 1\|253 | 40\|9228 | 0.6718767 | 0.9442592 | ENSG00000188257 | PLA2G2A | hsa:5320 | 1 | 0 |  | 1 | ENSG00000188257 |
| hsa04620 | Toll-like receptor signaling pathway | 2\|253 | 85\|9228 | 0.6816652 | 0.9515812 | ENSG00000169429/ENSG00000137462 | CXCL8/TLR2 | hsa:3576/hsa:7097 | 2 | 0 |  | 2 | ENSG00000169429/ENSG00000137462 |
| hsa04136 | Autophagy - other | 1\|253 | 42\|9228 | 0.6896992 | 0.9515812 | ENSG00000139112 | GABARAPL1 | hsa:23710 | 1 | 0 |  | 1 | ENSG00000139112 |
| hsa04010 | MAPK signaling pathway | 8\|253 | 331\|9228 | 0.6924849 | 0.9515812 | ENSG00000160867/ENSG00000152689/ENSG00000128052/ENSG00000130829/ENSG00000092969/ENSG00000109321/ENSG00000037280/ENSG00000120875 | FGFR4/RASGRP3/KDR/DUSP9/TGFB2/AREG/FLT4/DUSP4 | hsa:2264/hsa:25780/hsa:3791/hsa:1852/hsa:7042/hsa:374/hsa:2324/hsa:1846 | 8 | 3 | ENSG00000160867/ENSG00000130829/ENSG00000037280 | 5 | ENSG00000152689/ENSG00000128052/ENSG00000092969/ENSG00000109321/ENSG00000120875 |
| hsa05214 | Glioma | 2\|253 | 87\|9228 | 0.6939808 | 0.9515812 | novel.507/ENSG00000101412 | -/E2F1 | hsa:1870/hsa:1869 | 2 | 2 | novel.507/ENSG00000101412 | 0 |  |
| hsa04971 | Gastric acid secretion | 2\|253 | 88\|9228 | 0.6999903 | 0.9515812 | ENSG00000164742/ENSG00000147606 | ADCY1/SLC26A7 | hsa:107/hsa:115111 | 2 | 1 | ENSG00000164742 | 1 | ENSG00000147606 |
| hsa05160 | Hepatitis C | 4\|253 | 173\|9228 | 0.7033354 | 0.9515812 | novel.507/ENSG00000101412/ENSG00000111335/ENSG00000134321 | -/E2F1/OAS2/RSAD2 | hsa:1870/hsa:1869/hsa:4939/hsa:91543 | 4 | 2 | novel.507/ENSG00000101412 | 2 | ENSG00000111335/ENSG00000134321 |
| hsa05216 | Thyroid cancer | 1\|253 | 44\|9228 | 0.7065573 | 0.9515812 | ENSG00000132170 | PPARG | hsa:5468 | 1 | 0 |  | 1 | ENSG00000132170 |
| hsa04659 | Th17 cell differentiation | 2\|253 | 91\|9228 | 0.7174349 | 0.9515812 | ENSG00000131759/ENSG00000104998 | RARA/IL27RA | hsa:5914/hsa:9466 | 2 | 2 | ENSG00000131759/ENSG00000104998 | 0 |  |
| hsa05120 | Epithelial cell signaling in Helicobacter pylori infection | 2\|253 | 91\|9228 | 0.7174349 | 0.9515812 | ENSG00000169429/ENSG00000081041 | CXCL8/CXCL2 | hsa:3576/hsa:2920 | 2 | 0 |  | 2 | ENSG00000169429/ENSG00000081041 |
| hsa00620 | Pyruvate metabolism | 1\|253 | 46\|9228 | 0.7225029 | 0.9515812 | ENSG00000137124 | ALDH1B1 | hsa:219 | 1 | 1 | ENSG00000137124 | 0 |  |
| hsa04137 | Mitophagy - animal | 4\|253 | 180\|9228 | 0.7323279 | 0.9515812 | ENSG00000254337/ENSG00000101412/ENSG00000139112/ENSG00000123240 | AC083967.1/E2F1/GABARAPL1/OPTN | hsa:55626/hsa:1869/hsa:23710/hsa:10133 | 4 | 2 | ENSG00000254337/ENSG00000101412 | 2 | ENSG00000139112/ENSG00000123240 |
| hsa04750 | Inflammatory mediator regulation of TRP channels | 2\|253 | 96\|9228 | 0.7446160 | 0.9515812 | ENSG00000164742/ENSG00000168398 | ADCY1/BDKRB2 | hsa:107/hsa:624 | 2 | 1 | ENSG00000164742 | 1 | ENSG00000168398 |
| hsa00071 | Fatty acid degradation | 1\|253 | 49\|9228 | 0.7448175 | 0.9515812 | ENSG00000137124 | ALDH1B1 | hsa:219 | 1 | 1 | ENSG00000137124 | 0 |  |
| hsa01521 | EGFR tyrosine kinase inhibitor resistance | 2\|253 | 97\|9228 | 0.7497766 | 0.9515812 | ENSG00000128052/ENSG00000171791 | KDR/BCL2 | hsa:3791/hsa:596 | 2 | 1 | ENSG00000171791 | 1 | ENSG00000128052 |
| hsa00514 | Other types of O-glycan biosynthesis | 1\|253 | 50\|9228 | 0.7518510 | 0.9515812 | ENSG00000144278 | GALNT13 | hsa:114805 | 1 | 0 |  | 1 | ENSG00000144278 |
| hsa05230 | Central carbon metabolism in cancer | 2\|253 | 98\|9228 | 0.7548476 | 0.9515812 | ENSG00000141526/ENSG00000117394 | SLC16A3/SLC2A1 | hsa:9123/hsa:6513 | 2 | 2 | ENSG00000141526/ENSG00000117394 | 0 |  |
| hsa00565 | Ether lipid metabolism | 1\|253 | 51\|9228 | 0.7586915 | 0.9515812 | ENSG00000188257 | PLA2G2A | hsa:5320 | 1 | 0 |  | 1 | ENSG00000188257 |
| hsa04550 | Signaling pathways regulating pluripotency of stem cells | 3\|253 | 144\|9228 | 0.7605231 | 0.9515812 | ENSG00000160867/ENSG00000130829/ENSG00000105991 | FGFR4/DUSP9/HOXA1 | hsa:2264/hsa:1852/hsa:3198 | 3 | 2 | ENSG00000160867/ENSG00000130829 | 1 | ENSG00000105991 |
| hsa04722 | Neurotrophin signaling pathway | 3\|253 | 145\|9228 | 0.7646354 | 0.9515812 | ENSG00000007264/ENSG00000111348/ENSG00000171791 | MATK/ARHGDIB/BCL2 | hsa:4145/hsa:397/hsa:596 | 3 | 2 | ENSG00000007264/ENSG00000171791 | 1 | ENSG00000111348 |
| hsa00970 | Aminoacyl-tRNA biosynthesis | 1\|253 | 52\|9228 | 0.7653441 | 0.9515812 | novel.282 |  | hsa:54938 | 1 | 0 |  | 1 | novel.282 |
| hsa05203 | Viral carcinogenesis | 6\|253 | 275\|9228 | 0.7707915 | 0.9515812 | ENSG00000157613/ENSG00000179388/ENSG00000117399/ENSG00000124635/ENSG00000145386/novel.600 | CREB3L1/EGR3/CDC20/HIST1H2BJ/CCNA2/- | hsa:90993/hsa:1960/hsa:991/hsa:8970/hsa:890/hsa:1388 | 6 | 5 | ENSG00000179388/ENSG00000117399/ENSG00000124635/ENSG00000145386/novel.600 | 1 | ENSG00000157613 |
| hsa04670 | Leukocyte transendothelial migration | 3\|253 | 147\|9228 | 0.7726886 | 0.9515812 | ENSG00000107562/ENSG00000179776/ENSG00000116701 | CXCL12/CDH5/NCF2 | hsa:6387/hsa:1003/hsa:4688 | 3 | 1 | ENSG00000107562 | 2 | ENSG00000179776/ENSG00000116701 |
| hsa05130 | Pathogenic Escherichia coli infection | 6\|253 | 276\|9228 | 0.7737628 | 0.9515812 | ENSG00000169429/ENSG00000214222/ENSG00000123416/ENSG00000109062/ENSG00000006453/ENSG00000104833 | CXCL8/TUBBP2/TUBA1B/SLC9A3R1/BAIAP2L1/TUBB4A | hsa:3576/hsa:203068/hsa:10376/hsa:9368/hsa:55971/hsa:10382 | 6 | 4 | ENSG00000214222/ENSG00000123416/ENSG00000109062/ENSG00000104833 | 2 | ENSG00000169429/ENSG00000006453 |
| hsa04919 | Thyroid hormone signaling pathway | 3\|253 | 148\|9228 | 0.7766303 | 0.9515812 | ENSG00000159200/ENSG00000117394/ENSG00000211448 | RCAN1/SLC2A1/DIO2 | hsa:1827/hsa:6513/hsa:1734 | 3 | 1 | ENSG00000117394 | 2 | ENSG00000159200/ENSG00000211448 |
| hsa00140 | Steroid hormone biosynthesis | 1\|253 | 54\|9228 | 0.7781062 | 0.9515812 | ENSG00000101846 | STS | hsa:412 | 1 | 0 |  | 1 | ENSG00000101846 |
| hsa00380 | Tryptophan metabolism | 1\|253 | 54\|9228 | 0.7781062 | 0.9515812 | ENSG00000137124 | ALDH1B1 | hsa:219 | 1 | 1 | ENSG00000137124 | 0 |  |
| hsa04520 | Adherens junction | 2\|253 | 103\|9228 | 0.7788948 | 0.9515812 | ENSG00000173482/ENSG00000127329 | PTPRM/PTPRB | hsa:5797/hsa:5787 | 2 | 0 |  | 2 | ENSG00000173482/ENSG00000127329 |
| hsa04912 | GnRH signaling pathway | 2\|253 | 104\|9228 | 0.7834499 | 0.9515812 | ENSG00000135346/ENSG00000164742 | CGA/ADCY1 | hsa:1081/hsa:107 | 2 | 1 | ENSG00000164742 | 1 | ENSG00000135346 |
| hsa00250 | Alanine, aspartate and glutamate metabolism | 1\|253 | 55\|9228 | 0.7842256 | 0.9515812 | ENSG00000086205 | FOLH1 | hsa:2346 | 1 | 0 |  | 1 | ENSG00000086205 |
| hsa04730 | Long-term depression | 1\|253 | 56\|9228 | 0.7901769 | 0.9515812 | ENSG00000185532 | PRKG1 | hsa:5592 | 1 | 0 |  | 1 | ENSG00000185532 |
| hsa05164 | Influenza A | 4\|253 | 196\|9228 | 0.7905444 | 0.9515812 | ENSG00000137648/ENSG00000169429/ENSG00000111335/ENSG00000134321 | TMPRSS4/CXCL8/OAS2/RSAD2 | hsa:56649/hsa:3576/hsa:4939/hsa:91543 | 4 | 0 |  | 4 | ENSG00000137648/ENSG00000169429/ENSG00000111335/ENSG00000134321 |
| hsa05412 | Arrhythmogenic right ventricular cardiomyopathy (ARVC) | 2\|253 | 107\|9228 | 0.7966258 | 0.9544825 | ENSG00000005961/ENSG00000115221 | ITGA2B/ITGB6 | hsa:3674/hsa:3694 | 2 | 1 | ENSG00000005961 | 1 | ENSG00000115221 |
| hsa04340 | Hedgehog signaling pathway | 1\|253 | 58\|9228 | 0.8015933 | 0.9560287 | ENSG00000171791 | BCL2 | hsa:596 | 1 | 1 | ENSG00000171791 | 0 |  |
| hsa04330 | Notch signaling pathway | 1\|253 | 60\|9228 | 0.8123909 | 0.9575123 | ENSG00000135547 | HEY2 | hsa:23493 | 1 | 1 | ENSG00000135547 | 0 |  |
| hsa04666 | Fc gamma R-mediated phagocytosis | 2\|253 | 112\|9228 | 0.8170181 | 0.9575123 | ENSG00000078053/ENSG00000277443 | AMPH/MARCKS | hsa:273/hsa:4082 | 2 | 0 |  | 2 | ENSG00000078053/ENSG00000277443 |
| hsa05100 | Bacterial invasion of epithelial cells | 2\|253 | 112\|9228 | 0.8170181 | 0.9575123 | ENSG00000197959/ENSG00000116670 | DNM3/MAD2L2 | hsa:26052/hsa:10459 | 2 | 1 | ENSG00000116670 | 1 | ENSG00000197959 |
| hsa04622 | RIG-I-like receptor signaling pathway | 1\|253 | 61\|9228 | 0.8175682 | 0.9575123 | ENSG00000169429 | CXCL8 | hsa:3576 | 1 | 0 |  | 1 | ENSG00000169429 |
| hsa05014 | Amyotrophic lateral sclerosis (ALS) | 1\|253 | 62\|9228 | 0.8226031 | 0.9590889 | ENSG00000171791 | BCL2 | hsa:596 | 1 | 1 | ENSG00000171791 | 0 |  |
| hsa04072 | Phospholipase D signaling pathway | 3\|253 | 165\|9228 | 0.8354447 | 0.9688755 | ENSG00000197959/ENSG00000169429/ENSG00000164742 | DNM3/CXCL8/ADCY1 | hsa:26052/hsa:3576/hsa:107 | 3 | 1 | ENSG00000164742 | 2 | ENSG00000197959/ENSG00000169429 |
| hsa04723 | Retrograde endocannabinoid signaling | 3\|253 | 166\|9228 | 0.8384500 | 0.9688755 | ENSG00000127588/ENSG00000187730/ENSG00000164742 | GNG13/GABRD/ADCY1 | hsa:51764/hsa:2563/hsa:107 | 3 | 3 | ENSG00000127588/ENSG00000187730/ENSG00000164742 | 0 |  |
| hsa04370 | VEGF signaling pathway | 1\|253 | 69\|9228 | 0.8541770 | 0.9740615 | ENSG00000128052 | KDR | hsa:3791 | 1 | 0 |  | 1 | ENSG00000128052 |
| hsa04924 | Renin secretion | 1\|253 | 69\|9228 | 0.8541770 | 0.9740615 | ENSG00000078401 | EDN1 | hsa:1906 | 1 | 0 |  | 1 | ENSG00000078401 |
| hsa05133 | Pertussis | 1\|253 | 69\|9228 | 0.8541770 | 0.9740615 | ENSG00000169429 | CXCL8 | hsa:3576 | 1 | 0 |  | 1 | ENSG00000169429 |
| hsa04720 | Long-term potentiation | 1\|253 | 72\|9228 | 0.8659318 | 0.9788115 | ENSG00000164742 | ADCY1 | hsa:107 | 1 | 1 | ENSG00000164742 | 0 |  |
| hsa04140 | Autophagy - animal | 5\|253 | 270\|9228 | 0.8680429 | 0.9788115 | ENSG00000254337/ENSG00000171791/ENSG00000139112/ENSG00000078804/ENSG00000062716 | AC083967.1/BCL2/GABARAPL1/TP53INP2/VMP1 | hsa:55626/hsa:596/hsa:23710/hsa:58476/hsa:81671 | 5 | 2 | ENSG00000254337/ENSG00000171791 | 3 | ENSG00000139112/ENSG00000078804/ENSG00000062716 |
| hsa04978 | Mineral absorption | 1\|253 | 73\|9228 | 0.8696364 | 0.9788115 | ENSG00000198417 | MT1F | hsa:4494 | 1 | 1 | ENSG00000198417 | 0 |  |
| hsa04921 | Oxytocin signaling pathway | 3\|253 | 180\|9228 | 0.8757989 | 0.9814988 | ENSG00000159200/ENSG00000116741/ENSG00000164742 | RCAN1/RGS2/ADCY1 | hsa:1827/hsa:5997/hsa:107 | 3 | 1 | ENSG00000164742 | 2 | ENSG00000159200/ENSG00000116741 |
| hsa04213 | Longevity regulating pathway - multiple species | 1\|253 | 77\|9228 | 0.8834626 | 0.9816251 | ENSG00000164742 | ADCY1 | hsa:107 | 1 | 1 | ENSG00000164742 | 0 |  |
| hsa04658 | Th1 and Th2 cell differentiation | 1\|253 | 77\|9228 | 0.8834626 | 0.9816251 | ENSG00000138378 | STAT4 | hsa:6775 | 1 | 0 |  | 1 | ENSG00000138378 |
| hsa05205 | Proteoglycans in cancer | 4\|253 | 238\|9228 | 0.8962298 | 0.9855332 | ENSG00000128052/ENSG00000092969/ENSG00000090447/ENSG00000137462 | KDR/TGFB2/TFAP4/TLR2 | hsa:3791/hsa:7042/hsa:7023/hsa:7097 | 4 | 1 | ENSG00000090447 | 3 | ENSG00000128052/ENSG00000092969/ENSG00000137462 |
| hsa05170 | Human immunodeficiency virus 1 infection | 4\|253 | 242\|9228 | 0.9033194 | 0.9855332 | ENSG00000127588/ENSG00000171791/ENSG00000137462/ENSG00000157456 | GNG13/BCL2/TLR2/CCNB2 | hsa:51764/hsa:596/hsa:7097/hsa:9133 | 4 | 3 | ENSG00000127588/ENSG00000171791/ENSG00000157456 | 1 | ENSG00000137462 |
| hsa04721 | Synaptic vesicle cycle | 1\|253 | 87\|9228 | 0.9119675 | 0.9855332 | ENSG00000197959 | DNM3 | hsa:26052 | 1 | 0 |  | 1 | ENSG00000197959 |
| hsa04144 | Endocytosis | 6\|253 | 343\|9228 | 0.9138867 | 0.9855332 | ENSG00000160867/ENSG00000078053/ENSG00000197959/ENSG00000153071/ENSG00000114423/ENSG00000024422 | FGFR4/AMPH/DNM3/DAB2/CBLB/EHD2 | hsa:2264/hsa:273/hsa:26052/hsa:1601/hsa:868/hsa:30846 | 6 | 2 | ENSG00000160867/ENSG00000024422 | 4 | ENSG00000078053/ENSG00000197959/ENSG00000153071/ENSG00000114423 |
| hsa04530 | Tight junction | 3\|253 | 200\|9228 | 0.9159061 | 0.9855332 | ENSG00000123416/ENSG00000166025/ENSG00000109062 | TUBA1B/AMOTL1/SLC9A3R1 | hsa:10376/hsa:154810/hsa:9368 | 3 | 3 | ENSG00000123416/ENSG00000166025/ENSG00000109062 | 0 |  |
| hsa04020 | Calcium signaling pathway | 3\|253 | 202\|9228 | 0.9191910 | 0.9855332 | ENSG00000168539/ENSG00000164742/ENSG00000168398 | CHRM1/ADCY1/BDKRB2 | hsa:1128/hsa:107/hsa:624 | 3 | 2 | ENSG00000168539/ENSG00000164742 | 1 | ENSG00000168398 |
| hsa00230 | Purine metabolism | 2\|253 | 153\|9228 | 0.9261110 | 0.9855332 | ENSG00000168032/ENSG00000164742 | ENTPD3/ADCY1 | hsa:956/hsa:107 | 2 | 1 | ENSG00000164742 | 1 | ENSG00000168032 |
| hsa04141 | Protein processing in endoplasmic reticulum | 3\|253 | 210\|9228 | 0.9311951 | 0.9855332 | ENSG00000162909/ENSG00000171791/novel.600 | CAPN2/BCL2/- | hsa:824/hsa:596/hsa:1388 | 3 | 2 | ENSG00000171791/novel.600 | 1 | ENSG00000162909 |
| hsa04660 | T cell receptor signaling pathway | 1\|253 | 98\|9228 | 0.9353628 | 0.9855332 | ENSG00000114423 | CBLB | hsa:868 | 1 | 0 |  | 1 | ENSG00000114423 |
| hsa05235 | PD-L1 expression and PD-1 checkpoint pathway in cancer | 1\|253 | 99\|9228 | 0.9371540 | 0.9855332 | ENSG00000137462 | TLR2 | hsa:7097 | 1 | 0 |  | 1 | ENSG00000137462 |
| hsa05132 | Salmonella infection | 5\|253 | 317\|9228 | 0.9394086 | 0.9855332 | ENSG00000169429/ENSG00000171791/ENSG00000023445/ENSG00000137462/ENSG00000233328 | CXCL8/BCL2/BIRC3/TLR2/PFN1P1 | hsa:3576/hsa:596/hsa:330/hsa:7097/hsa:5216 | 5 | 2 | ENSG00000171791/ENSG00000233328 | 3 | ENSG00000169429/ENSG00000023445/ENSG00000137462 |
| hsa04142 | Lysosome | 2\|253 | 162\|9228 | 0.9399240 | 0.9855332 | ENSG00000119899/ENSG00000102393 | SLC17A5/GLA | hsa:26503/hsa:2717 | 2 | 1 | ENSG00000102393 | 1 | ENSG00000119899 |
| hsa05135 | Yersinia infection | 2\|253 | 164\|9228 | 0.9426429 | 0.9855332 | ENSG00000169429/ENSG00000082074 | CXCL8/FYB1 | hsa:3576/hsa:2533 | 2 | 0 |  | 2 | ENSG00000169429/ENSG00000082074 |
| hsa04350 | TGF-beta signaling pathway | 1\|253 | 103\|9228 | 0.9438376 | 0.9855332 | ENSG00000092969 | TGFB2 | hsa:7042 | 1 | 0 |  | 1 | ENSG00000092969 |
| hsa04726 | Serotonergic synapse | 1\|253 | 103\|9228 | 0.9438376 | 0.9855332 | ENSG00000127588 | GNG13 | hsa:51764 | 1 | 1 | ENSG00000127588 | 0 |  |
| hsa04310 | Wnt signaling pathway | 2\|253 | 169\|9228 | 0.9489388 | 0.9865448 | ENSG00000133067/ENSG00000175792 | LGR6/RUVBL1 | hsa:59352/hsa:8607 | 2 | 1 | ENSG00000175792 | 1 | ENSG00000133067 |
| hsa04910 | Insulin signaling pathway | 2\|253 | 172\|9228 | 0.9523952 | 0.9865448 | ENSG00000109819/ENSG00000114423 | PPARGC1A/CBLB | hsa:10891/hsa:868 | 2 | 0 |  | 2 | ENSG00000109819/ENSG00000114423 |
| hsa01200 | Carbon metabolism | 2\|253 | 183\|9228 | 0.9632569 | 0.9911362 | ENSG00000186197/ENSG00000092621 | EDARADD/PHGDH | hsa:2023/hsa:26227 | 2 | 1 | ENSG00000092621 | 1 | ENSG00000186197 |
| hsa03013 | RNA transport | 3\|253 | 246\|9228 | 0.9674757 | 0.9911362 | novel.674/ENSG00000013810/ENSG00000183684 | -/TACC3/ALYREF | hsa:79897/hsa:10460/hsa:10189 | 3 | 3 | novel.674/ENSG00000013810/ENSG00000183684 | 0 |  |
| hsa03015 | mRNA surveillance pathway | 1\|253 | 126\|9228 | 0.9706072 | 0.9911362 | ENSG00000183684 | ALYREF | hsa:10189 | 1 | 1 | ENSG00000183684 | 0 |  |
| hsa04714 | Thermogenesis | 5\|253 | 360\|9228 | 0.9720759 | 0.9911362 | ENSG00000185532/ENSG00000157613/ENSG00000109819/ENSG00000164742/ENSG00000132170 | PRKG1/CREB3L1/PPARGC1A/ADCY1/PPARG | hsa:5592/hsa:90993/hsa:10891/hsa:107/hsa:5468 | 5 | 1 | ENSG00000164742 | 4 | ENSG00000185532/ENSG00000157613/ENSG00000109819/ENSG00000132170 |
| hsa05131 | Shigellosis | 5\|253 | 375\|9228 | 0.9789442 | 0.9942402 | ENSG00000169429/ENSG00000162909/ENSG00000171791/ENSG00000139112/ENSG00000233328 | CXCL8/CAPN2/BCL2/GABARAPL1/PFN1P1 | hsa:3576/hsa:824/hsa:596/hsa:23710/hsa:5216 | 5 | 2 | ENSG00000171791/ENSG00000233328 | 3 | ENSG00000169429/ENSG00000162909/ENSG00000139112 |
| hsa03040 | Spliceosome | 2\|253 | 229\|9228 | 0.9879165 | 0.9988420 | ENSG00000130520/ENSG00000183684 | LSM4/ALYREF | hsa:25804/hsa:10189 | 2 | 2 | ENSG00000130520/ENSG00000183684 | 0 |  |
| hsa05012 | Parkinson disease | 4\|253 | 430\|9228 | 0.9979242 | 0.9988420 | ENSG00000214222/ENSG00000123416/ENSG00000180176/ENSG00000104833 | TUBBP2/TUBA1B/TH/TUBB4A | hsa:203068/hsa:10376/hsa:7054/hsa:10382 | 4 | 4 | ENSG00000214222/ENSG00000123416/ENSG00000180176/ENSG00000104833 | 0 |  |
| hsa04932 | Non-alcoholic fatty liver disease (NAFLD) | 1\|253 | 225\|9228 | 0.9982235 | 0.9988420 | ENSG00000169429 | CXCL8 | hsa:3576 | 1 | 0 |  | 1 | ENSG00000169429 |
| hsa05017 | Spinocerebellar ataxia | 1\|253 | 240\|9228 | 0.9988420 | 0.9988420 | ENSG00000254337 | AC083967.1 | hsa:55626 | 1 | 1 | ENSG00000254337 | 0 |  |
